# Differential proteomic profile of lumbar and ventricular cerebrospinal fluid

**DOI:** 10.1186/s12987-022-00405-0

**Published:** 2023-01-21

**Authors:** Nina Rostgaard, Markus Harboe Olsen, Maud Ottenheijm, Lylia Drici, Anja Hviid Simonsen, Peter Plomgaard, Hanne Gredal, Helle Harding Poulsen, Henrik Zetterberg, Kaj Blennow, Steen Gregers Hasselbalch, Nanna MacAulay, Marianne Juhler

**Affiliations:** 1 Department of Neurosurgery, The Neuroscience Centre, Copenhagen University Hospital - Rigshospitalet, Copenhagen, Denmark; 2 Department of Neuroanaesthesiology, The Neuroscience Centre, Copenhagen University Hospital - Rigshospitalet, Copenhagen, Denmark; 3 NNF Center for Protein Research, University of Copenhagen, Copenhagen, Denmark; 4 Department of Clinical Biochemistry, Copenhagen University Hospital - Rigshospitalet, Copenhagen, Denmark; 5 Danish Dementia Research Centre, Department of Neurology, Neuroscience Centre, Copenhagen University Hospital - Rigshospitalet, Copenhagen, Denmark; 6 Department of Veterinary Clinical Sciences, University of Copenhagen, Copenhagen, Denmark; 7 Department of Psychiatry and Neurochemistry, Institute of Neuroscience and Physiology, The Sahlgrenska Academy at the University of Gothenburg, Mölndal, Gothenburg, Sweden; 8 Clinical Neurochemistry Laboratory, Sahlgrenska University Hospital, Mölndal, Gothenburg, Sweden; 9 Department of Neurodegenerative Disease, UCL Institute of Neurology, Queen Square, London, UK; 10 UK Dementia Research Institute at UCL, London, UK; 11 Hong Kong Center for Neurodegenerative Diseases, Clear Water Bay, Hong Kong, China; 12 Department of Clinical Medicine, Faculty of Health and Medical Sciences, University of Copenhagen, Copenhagen, Denmark; 13 Department of Neuroscience, University of Copenhagen, Copenhagen, Denmark

**Keywords:** Cerebrospinal fluid, Biomarkers, Mass spectrometry, Proteomics, Idiopathic normal pressure hydrocephalus

## Abstract

**Background:**

Pathological cerebral conditions may manifest in altered composition of the cerebrospinal fluid (CSF). Although diagnostic CSF analysis seeks to establish pathological disturbances in the brain proper, CSF is generally sampled from the lumbar compartment for reasons of technical ease and ethical considerations. We here aimed to compare the molecular composition of CSF obtained from the ventricular versus the lumbar CSF compartments to establish a relevance for employing lumbar CSF as a proxy for the CSF bathing the brain tissue.

**Methods:**

CSF was collected from 46 patients with idiopathic normal pressure hydrocephalus (iNPH) patients during their diagnostic workup (lumbar samples) and in connection with their subsequent CSF diversion shunt surgery (ventricular samples). The mass-spectrometry-based proteomic profile was determined in these samples and in addition, selected biomarkers were quantified with ELISA (S100B, neurofilament light (NfL), amyloid-β (Aβ_40_, Aβ_42_), and total tau (T-tau) and phosphorylated tau (P-tau) forms). The latter analysis was extended to include paired porcine samples obtained from the lumbar compartment and the cerebromedullary cistern closely related to the ventricles.

**Results:**

In total 1231 proteins were detected in the human CSF. Of these, 216 distributed equally in the two CSF compartments, whereas 22 were preferentially (or solely) present in the ventricular CSF and four in the lumbar CSF. The selected biomarkers of neurodegeneration and Alzheimer’s disease displayed differential distribution, some with higher (S100B, T-tau, and P-tau) and some with lower (NfL, Aβ_40_, Aβ_42_) levels in the ventricular compartment. In the porcine samples, all biomarkers were most abundant in the lumbar CSF.

**Conclusions:**

The overall proteomic profile differs between the ventricular and the lumbar CSF compartments, and so does the distribution of clinically employed biomarkers. However, for a range of CSF proteins and biomarkers, one can reliably employ lumbar CSF as a proxy for ventricular CSF if or a lumbar/cranial index for the particular molecule has been established. It is therefore important to verify the compartmental preference of the proteins or biomarkers of interest prior to extrapolating from lumbar CSF to that of the ventricular fluid bordering the brain.

**Supplementary Information:**

The online version contains supplementary material available at 10.1186/s12987-022-00405-0.

## Introduction

Cerebrospinal fluid (CSF) surrounds the brain and fills the central ventricles and is thus in direct contact with the brain. The molecular composition of CSF may therefore reflect biochemical changes in intracranial fluid compartments, e.g., CSF and/or interstitial fluid. Accordingly, CSF analysis is commonly used as a surrogate measure for pathological conditions in the brain, and proteins detected in the CSF are used as biomarkers of disease both as a screening tool and as a supplementary information to diagnostic investigations [1–6]. CSF used for diagnostic workup is routinely accessed through lumbar puncture, which is performed widely on several clinical indications, e.g., Alzheimer’s disease [7–9], multiple sclerosis [10], neuroborreliosis [11–13], and Creutzfeldt-Jakob’s disease [14–16]. Although diagnostic CSF analysis seeks to establish pathological disturbances in the brain, CSF is generally sampled from the lumbar compartment for reasons of technical ease and ethical considerations, rather than from the ventricular compartment, which is envisaged to reflect the brain pathology more correctly.

The lumbar CSF compartment is anatomically remote from the ventricular compartment. This distance results in a gradient of protein concentration along the neuroaxis; the CSF total protein, albumin and IgG content increase progressively from the ventricles to the lumbar sac with a 60% higher protein content in lumbar CSF than in ventricular CSF in humans [17, 18] and canines [19]. These considerations refer to the general protein content in CSF, largely proteins derived from the blood, and do not necessarily reflect the CSF profile for specific proteins and various brain-derived biomarkers. Thus, it remains unresolved whether biomarkers detected in CSF obtained from the lumbar compartment are reliable indicators of intracranial pathology.

One of the difficulties in studying differences between brain and lumbar CSF is the limited access to ventricular CSF. Lumbar CSF can be collected during a wide range of diagnostic lumbar punctures and even from central nervous system (CNS)-healthy individuals, e.g., patients undergoing procedures with spinal anesthesia. However, it is generally only possible to collect human ventricular CSF from patients undergoing neurosurgery either as intracranial invasive diagnostic procedures or as treatment of neurological disorders. Thus, the CSF compartmental profile cannot be studied in CNS-healthy individuals. Idiopathic normal pressure hydrocephalus (iNPH) is a frequent neurological disease in the elderly population [20, 21], where lumbar CSF analysis for biomarkers of neurodegeneration is required for differential diagnosis against degenerative brain disease, in particular Alzheimer’s disease [22–24]. Patients with confirmed iNPH diagnosis are subsequently offered CSF diversion surgery that provides access to ventricular CSF during the surgical procedure. We therefore employed an iNPH patient group to determine the proteomic profile and biomarker levels in CSF obtained from the lumbar and ventricular compartments of the same patients, alongside a group of experimental pigs, in which CSF was sampled from the lumbar compartment and the cerebromedullar cistern near-simultaneously.

## Materials and methods

### Patients and sample collection

This study included CSF samples extracted from 46 iNPH patients [median age 75 (range 57–87) years; 21 F/25 M; mean body mass index (BMI) 28 (range 20–36)]. Patients were diagnosed with iNPH at the Danish Dementia Research Centre, Copenhagen University Hospital—Rigshospitalet, Denmark, according to the international guidelines from 2005 [25], including evaluation of cognitive impairment, gait/balance disturbances, urinary incontinence and brain imaging. All patients had a supplementary diagnostic test [infusion test followed by a tap-test using the CELDA system (Likvor, Umea, Sweden) through two lumbar needles]. The infusion test measures resting intracranial pressure (ICP) and resistance to outflow (R_out_). An abnormally high R_out_ and/or improved gait function following tap-test increase the diagnostic accuracy, but normal tests do not preclude iNPH [26]. The lumbar CSF sample was obtained from the infusion test during the diagnostic examination. The lumbar CSF samples were collected prior to Ringer infusion and the volume was replaced by Ringer solution prior to the lumbar infusion test. Ventricular CSF was collected upon ventriculo peritoneal (VP) shunt insertion (n = 39), endoscopic third ventriculostomy (ETV) upon indication of membraneous occlusion of the aquaduct (n = 1), tap test through shunt chamber (n = 3) or insertion of extraventricular drain (EVD) for diagnostic purposes including ICP monitoring and CSF dynamic investigation due to unclear tap test results (n = 3), according to standard procedures at the Department of Neurosurgery at Rigshospitalet, Copenhagen, Denmark. All patients were under general anesthesia during the ventricular sample collection apart from the 3 in whom samples were collected through the shunt chamber where no anesthesia was applied. The paired ventricular samples were collected within a median of 86 days (range 32–1355) after the lumbar CSF samples. The CSF samples were centrifuged at 2000×*g* for 10 min immediately after sampling and aliquoted in polypropylene microtubes (Sarstedt) before storage at − 80 °C [27]. Written informed consent was obtained from all patients and the study was approved by the Danish National Committee on Health Research Ethics (Approval No. H-19001474 and H-18046630) and the Danish Data Protection Agency (VD-2019-210).

### Animals

Danish mixed breeds of Yorkshire, Danish Landrace and Duroc pigs (n = 18) with a mean weight of 24.02 ± 4.37 kg and an estimated age of 10–13 weeks were included. CSF collection was performed with the pigs placed in lateral recumbency under general anaesthesia. Anaesthesia was induced with an intravenous injection of propofol (Propo Vet Multidose 10 mg/ml, Zoetis, Finland, 1–3 mg/kg) and subsequently maintained with isoflurane (IsoFlo Vet, Zoetis, Finland), 1–2.5 vol% inhalation in a circle system after an intramuscular premedication with 1 ml/10 kg of a custom-made Zoletil 50 Vet-mixture (125 mg zolazepam, 125 mg tiletamine dry matter (Zoletil50 Vet, Virbac, Denmark) dissolved in 6.25 ml xylazine (20 mg/ml, Rompun Vet, Elanco, Denmark), 2.5 ml ketamine (50 mg/ml, Ketaminol Vet, MSD Animal Health, Denmark) and 2.5 ml butorphanol (10 mg/ml, Torbugesic Vet, Zoetis, Finland)). Additional 0.07 ml acepromazine (10 mg/ml, Plegicil Vet, Pharmaxim, Sweden) and 0.5 ml methadone (10 mg/ml, Comfortan Vet., Dechra, Denmark) were administrated intramuscularly at the time of premedication. Upon shaving of all puncture sites and cleaning with 70% ethanol, 1 ml CSF was collected from cisterna magna (the atlantooccipital site) and from the lumbar cistern (the lumbar site between L7 and S1) with 90 mm and 75 mm spinal needles, respectively. Both CSF samples were obtained with < 5 min interval approximately 3 h after induction of anesthesia. Animals were excluded if puncturing failed at one site. To avoid bias, the order of CSF collection was randomly controlled for each animal. All animal experiments were performed on pigs employed for veterinary student training of abdominal surgical procedures and according to the legislation for animal protection and care, animal permission no. 2016-15-0201-00957 approved by the Danish Animal Experiments Inspectorate.

### Protein digestion and Evotips loading

Human CSF sample preparation was performed on an Agilent Bravo Liquid Handling Platform (Agilent) according to an optimized version of previously published protocols [28, 29]. Briefly, CSF samples were aliquoted into a 96-well format plate and introduced to the Bravo Robot (Agilent). 20 µl CSF sample was mixed with 30 µl PreOmics Lysis buffer (P.O. 00001, PreOmics GmbH) and incubated at 95 °C for 10 min in order to denature proteins, reduce disulfide bridges and alkylate cysteines [30]. After cooling the sample for 15 min at room temperature, trypsin and LysC (0.5 µg/ul, Promega) were added in a ratio of 1 µg enzyme to 100 µg proteins and the mixture incubated at 37 °C for 4 h. The peptide mixtures were diluted in 100 µl 99% isopropanol, 1% Trifluoro-acetic acid (TFA) and desalted using two-gauge reversed-phase styrenedivinylbenzene (SDB-RPS) stage-tips. Afterwards, the stage-tips were washed using 200 µl 99% isopropanol, 1% TFA, followed by 200 µl 0.2% TFA. The purified peptides were eluted using 80% acetonitrile (VWR chemicals) containing 1% ammonia (Merck) and subsequently dried down. Peptides were resuspended in solvent A (0.1% formic acid (FA) in water) and loaded onto Evotips (Evosep Biosystem, Denmark) according to the manufacturer’s recommendations. The Evotips were wetted with isopropanol for 5 min, activated with 20 µl solvent B (99% ACN, 0.1% FA) and centrifuged at 700×*g* for 1 min. 20 µl of solvent A was then added to equilibrate the tips followed by sample loading. Finally, 20 µl solvent A was used to wash the Evotip and 100 µl was added to avoid drying.

### Liquid chromatography (LC) and mass spectrometry (MS) analysis

The samples were injected into an Exploris 480 Thermo Fischer Scientific system using Evosep One (Evosep Biosystem). A preset chromatographic method was used corresponding to 60 samples per day**.** The peptides were separated on an 8 cm Pepsep column (150 μm, ID 1.5 μm bead size Reprosil-Pur C18 beads, Marslev, Denmark) at 1 μl/min flow rate with a 21 min gradient. The heated capillary temperature was set to 275 °C, the spray voltage to 2650 V and the funnel radiofrequency to 40 Hz. The mass spectrometer was operated in a data-independent mode (DIA) with a full MS range from 350 to 1650 m/z at a resolution of 60,000 at 200 m/z. The AGC target was set to 300% with an injection time of 50 ms. The AGC value of the targeted MS2 experiment was set to 1000%. Thirty-two windows of variable sizes were defined for target MS2 (tMS2) acquisition and subjected to high-energy collisional dissociation (HCD) fragmentation with a normalized collision energy at 30%. Each tMS2 scan was acquired at a resolution of 30,000 with a maximum ion injection time (IT) of 100 ms for a scan range of m/z 349.5–1650.5.

### Data handling

The MS raw files were processed with Spectronaut version 15 (Biognosys, Switzerland). A previously generated CSF spectral library was imported from MaxQuant software analyses. The library contained 2733 protein groups and 17,301 peptides. DIA files were searched against the library using default parameters except for the normalization, which was set to local. Dynamic mass and retention time tolerances (for both MS1 and MS2) were applied. Q-value cutoff was set to 1% both at precursor and protein level using a mutated decoy method [31]. The calibration was performed based on a local regression model [32]. Protein data was exported from Spectronaut and further processed using the clinical knowledge graph (CKG) [33] together with their matching experimental and clinical data. Intensities were log-transformed before further statistical analysis. As the proteomic data are dependent on the amount of sample injected into the mass spectrometer we normalized (in-silico) the total protein intensity by sample. Similar approach has been reported previously [28].

### ELISA analyses

The CSF samples were analyzed at the Clinical Neurochemistry Laboratory, Sahlgrenska University Hospital, Mölndal, Sweden. CSF concentrations of Aβ_40_, Aβ_42_, total tau (T-tau) and phosphorylated tau (P-tau) were measured with Lumipulse technology (Fujirebio, Ghent, Belgium), as previously described [34]. CSF neurofilament light (NfL) concentration was measured using an in-house enzyme-linked immunosorbent assay as previously described [35]. S100B was analyzed with a Sangtec 100 ELISA kit (Diasorim, MN, USA). All biomarker measurements were performed using the same batch of reagents by board-certified laboratory technicians blinded to the clinical information. Lumbar and ventricular samples from each patient were run on the same plate to avoid plate-to-plate variance.

### Statistical analysis

Statistical analyses were carried out using R v. 4.1.0 (R Core Team, Vienna, Austria). Continuous data were presented as mean and standard deviation (SD) or median and interquartile range (IQR)/range depending on normality, while categorical data were presented as n, proportion, and percentage. Proteomics data were assessed for availability in both lumbar and ventricular CSF, to assess which proteins are available (above level of detection) in each compartment. A principal component analysis (PCA) plot with complete case analysis (those proteins available in all samples), and a PCA plot where missing data was imputed for proteins available for at least 10 pairs (available in both lumbar and ventricular CSF in at least 10 patients) were conducted [36]. PCA addresses if the patterns in the groups differ. For samples with at least 10 pairs, a Volcano plot reveals whether the levels are similar, higher lumbar, higher ventricular or shows a tendency towards one or the other. Proteins higher in each compartment require a difference in the Bonferroni-corrected P value of < 0.05 and two-fold change in concentration, while similar must be below a two-fold change and an unadjusted P value ≥ 0.05. The ELISA data were compared using Wilcoxon signed rank test with continuity correction for paired samples of lumbar and ventricular CSF after visual assessment of normality of the difference between lumbar and ventricular CSF (Additional file 1: Fig. S1). Furthermore, Pearson’s correlation coefficients were calculated to investigate the influence of the days between samples, age, and BMI (the latter in Additional file 1: Fig. S2). Non-normal distributed data were log transformed, and outliers were removed by identifying samples which were four times above the mean Cook’s distance [37]. Potential blood contamination in the CSF samples was assessed by quantification of blood proteins (Additional file 1: Fig. S3).

## Results

### Distinct proteomic profile of lumbar versus ventricular CSF

**Fig. 1:**
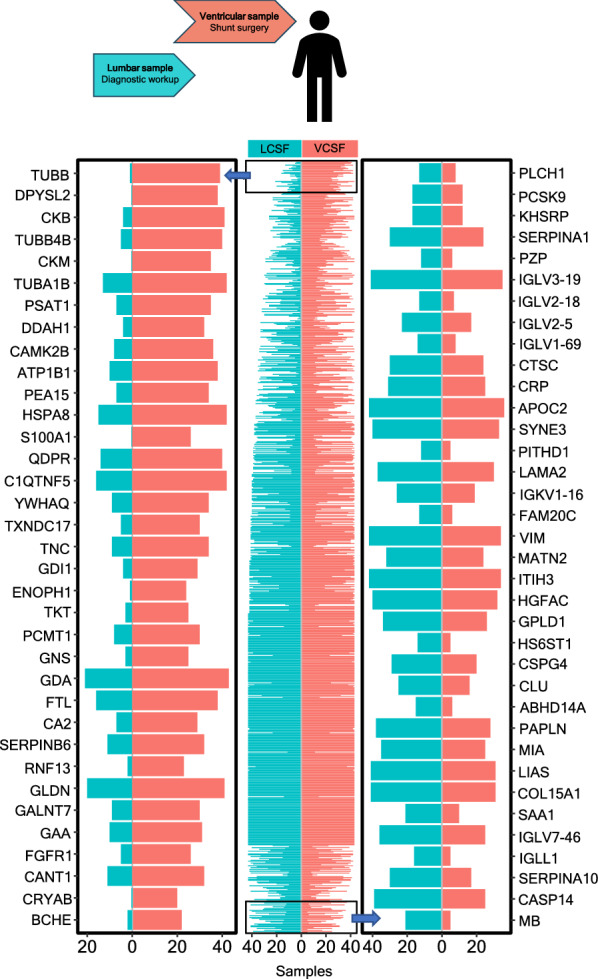
Proteomic profiles of lumbar versus ventricular CSF. Visualization of compartmentalized presence of different proteins detected by untargeted mass spectrometry of CSF obtained from lumbar and ventricular compartments of iNPH patients (n = 46). Left column represents a close-up of the 35 proteins detected in a higher number of ventricular samples than lumbar samples. Right column displays the top 35 proteins detected in a higher number of lumbar samples than ventricular samples. Red bars: ventricular CSF (VCSF); blue bars: lumbar CSF (LCSF)

**Fig. 2:**
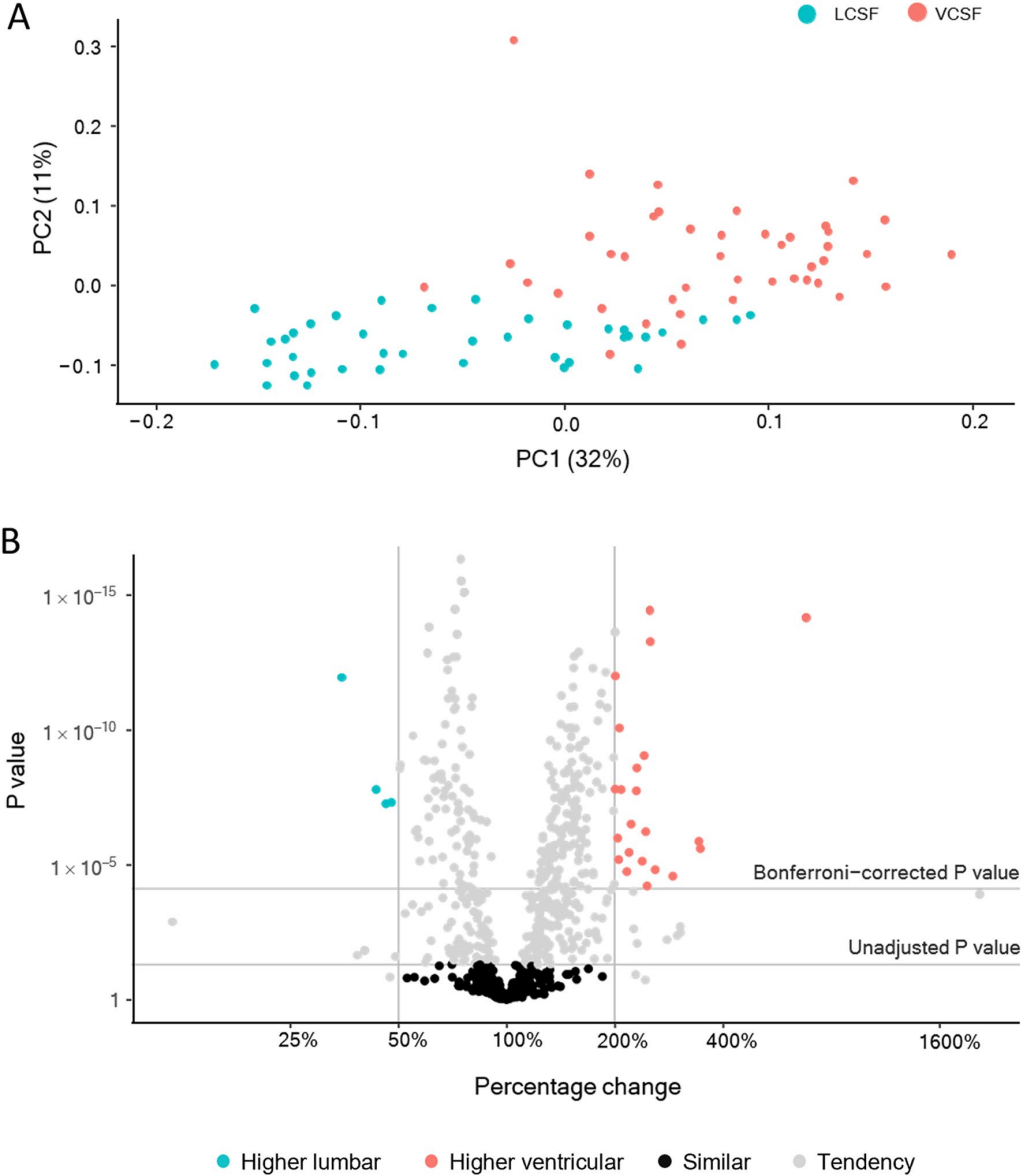
Distinct distribution of CSF proteins in lumbar and ventricular CSF. **A** PCA analysis of the CSF samples from the two compartments. **B** Volcano plot of proteomic data obtained from lumbar CSF (LCSF) compared to ventricular CSF (VCSF). The threshold of percentage changes was set to 200% increase compared to the other CSF pool (marked with vertical lines) and P < 0.05 after Bonferroni correction (marked with horizontal line), n = 46

To determine the proteomic profile of lumbar versus ventricular CSF, we employed lumbar samples obtained from 46 iNPH patients during their diagnostic work-up paired to ventricular samples obtained at subsequent neurosurgical procedure (i.e., shunt implantation, ETV, tap test or insertion of EVD). Mass spectrometry-based proteomic analysis of these samples identified 1231 unique proteins in either sample (see Additional file 2 for the complete list of detected proteins). Of those, 849 were detected in at least five samples in either compartment, 16 proteins were detected exclusively in the ventricular CSF, while no proteins were detected in only the lumbar compartment (Additional file 2). To visualize compartmentalized presence of the different proteins, the number of samples (lumbar versus ventricular) in which the protein was detected are illustrated in Fig. 1, middle column. The left column represents a close-up of the top 35 proteins detected in a higher number of ventricular samples than lumbar samples (out of 173 proteins, Additional file 2), whereas the right column displays the top 35 proteins detected in a higher number of lumbar samples than ventricular samples (Fig. 1). The PCA plot revealed two distinct proteomic profiles when comparing lumbar and ventricular CSF, suggesting an overall difference between the two CSF compartments (Fig. 2A). To determine the compartmentalized levels of the CSF proteins, the data was analyzed using paired t-test (result shown as volcano plot, Fig. 2B). To allow for paired comparison, only proteins detected in both compartments in at least 10 patients were included. Figure 2B reveals that 22 proteins were detected at significantly higher levels in ventricular CSF (> 200% and P < 0.05 after Bonferroni correction), while four proteins were detected at significantly higher levels in lumbar CSF (> 200% and P < 0.05 after Bonferroni correction). 216 proteins were identified at similar levels in the two CSF compartments and 425 showed a pattern of tendency to compartmentalize but did either not meet the chosen level change (less than twofold difference between the two compartments) or only reached significance (P < 0.05) without Bonferroni correction (Fig. 2B and Additional file 2).

### Clinical biomarkers distribute differentially in the CSF compartments

**Fig. 3:**
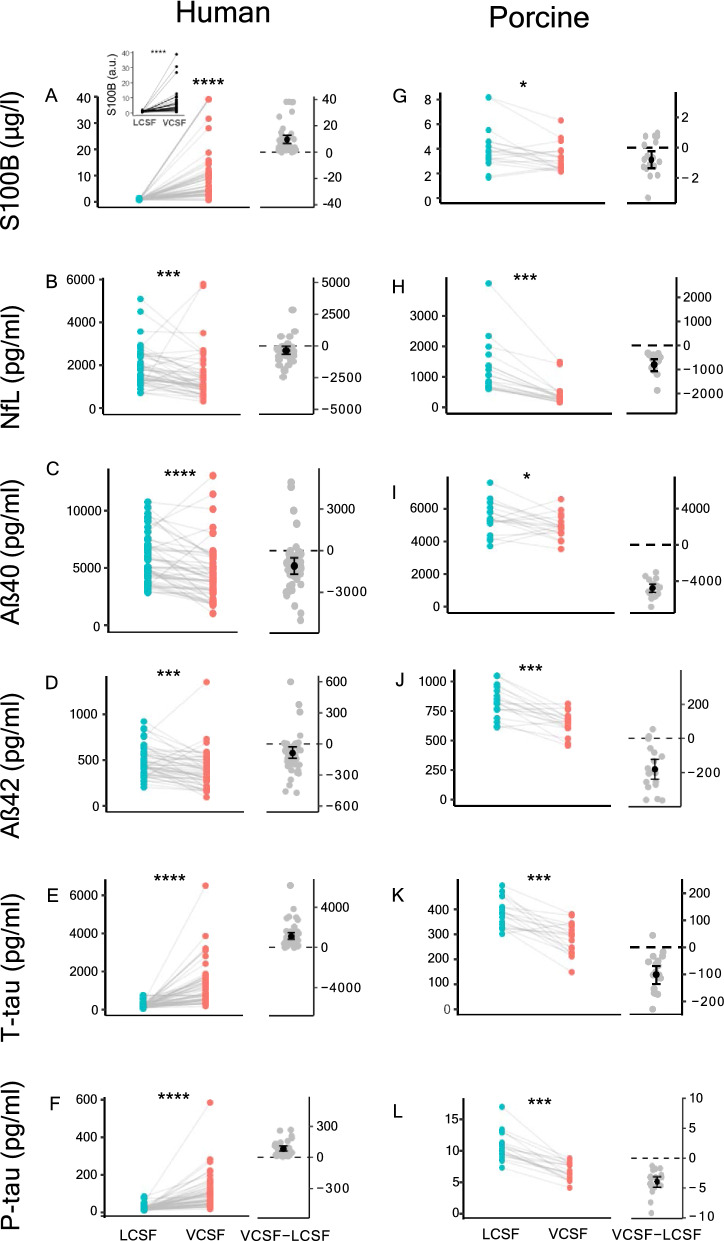
Quantitative assessment of CSF biomarkers by ELISA. **A-F** Levels of S100B, NfL, Ab40, Ab42, P-tau and T-tau in the human CSF samples, n = 46. The right part of the graphs represents the difference between the ventricular and the lumbar CSF concentrations in each patient. **H–L** Same biomarkers as in **A**–**F** quantified on porcine CSF samples, n = 18. The data were evaluated for statistical significance with Wilcoxon signed rank test. *P < 0.05, ***P < 0.001, ****P < 0.0001

**Fig. 4:**
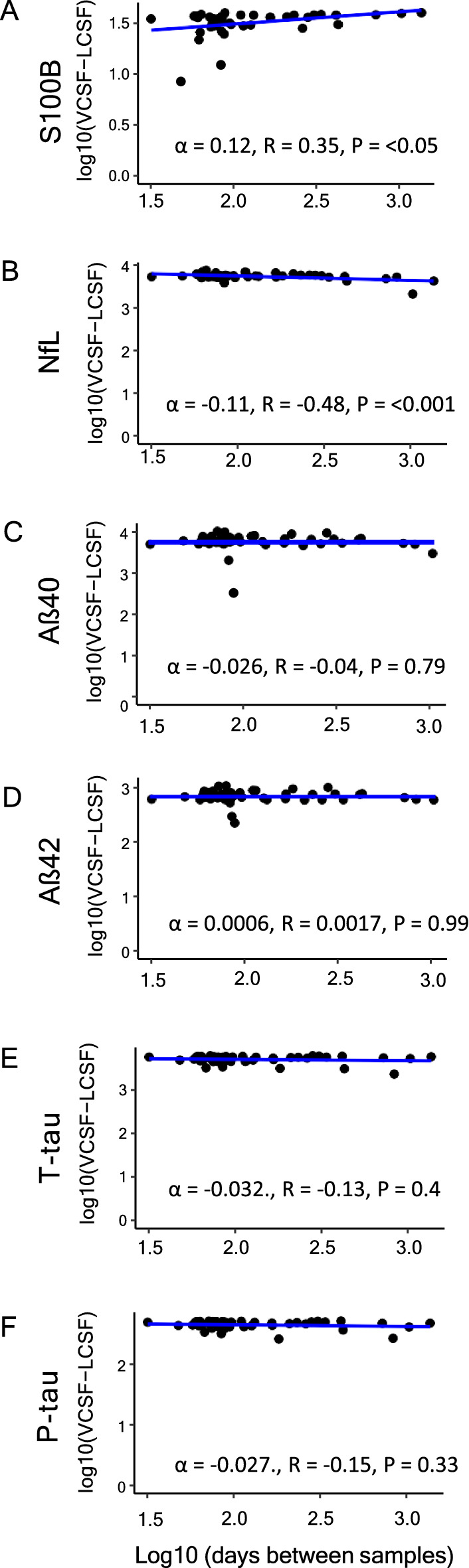
Correlation analysis of time between samples and biomarker levels. Correlation analysis of the difference in biomarker levels (lumbar subtracted from ventricular) versus the time interval between the sampling, n = 46. α; slope

To obtain quantitative insight into the distribution of clinically employed CSF biomarkers, we determined the ventricular versus lumbar levels of selected biomarkers with an ELISA-based approach. Of the employed biomarkers, only one (a biomarker of general neurodegeneration; S100B [38–41]) was detected in both the ELISA-based approach and the mass spectrometry-based analysis, and to verify the comparability between the approaches, we compared S100B between the methods (Fig. 3A). The ELISA analysis demonstrated significantly elevated S100B levels in the ventricular compartment (10.6 ± 10.2 vs. 0.95 ± 0.16 µg/l in the lumbar compartment, n = 46, P < 0.001, Fig. 3A), which aligned with the mass spectrometry-based analysis (7.14 ± 8.66 vs 0.68 ± 0.30 a.u.; n = 43, P < 0.001, Fig. 3A insert). Neurofilament light (NfL), another biomarker of general neurodegeneration [42–45], displayed the opposite pattern with a higher concentration in the lumbar compartment (1877 ± 927 vs 1512 ± 1162 pg/l in the ventricular compartment, n = 45, P < 0.001, Fig. 3B). Of four widely used Alzheimer’s disease biomarkers, Aβ_40_ and Aβ_42_ were more abundant in the lumbar CSF compartment (Aβ_40_: 5735 ± 2134 vs 4626 ± 2580 pg/l in the ventricular compartment, n = 46, P < 0.001, Fig. 3C; and Aβ_42_: 480 ± 167 vs 392 ± 207 pg/l in the ventricular compartment, n = 46, P < 0.01, Fig. 3D). In contrast, the other two established Alzheimer’s disease biomarkers, T-tau and P-tau were preferentially detected in the ventricular compartment (T-tau: 1336 ± 1182 vs 232 ± 160 pg/l in the lumbar compartment, n = 46, P < 0.001, Fig. 3E**;** and P-tau: 109 ± 96 vs 25 ± 15 pg/l in the lumbar compartment, n = 46, P < 0.001, Fig. 3F). The compartmental differences of these biomarkers could potentially arise with the interval between sampling of lumbar and ventricular CSF due to methodological issues (see Material and Methods). To reveal the potential influence of time between CSF sampling in the two compartments, we performed correlation analysis of the difference in CSF levels (lumbar CSF subtracted from ventricular CSF) versus the time interval between the sampling. The differential levels of the two biomarkers of general neurodegeneration displayed a slight time dependency, with a larger difference of S100B (n = 46, P < 0.05, Fig. 4A) and a reduced difference of NfL (n = 46, P < 0.001, Fig. 4B). However, time did not seem to influence the CSF levels of the Alzheimer’s disease biomarkers (Aβ_40_: P = 0.79, Fig. 4C; Aβ_42_: P = 0.99, Fig. 4D; T-tau: P = 0.40, Fig. 4E; P-tau: P = 0.33, Fig. 4F). Only Aβ_42_ decreased with increasing age (P < 0.05) and no other clinical biomarkers associated with age or BMI (Additional file 1: Fig. S2).

Curiously, all the six tested clinical biomarkers analyzed from near-simultaneously lumbar and cisternal sampling from the porcine model displayed higher levels in the lumbar compartment (S100B, P < 0.05, Fig. 3G; NfL, P < 0.001, Fig. 3H; Aβ_40_, P < 0.05, Fig. 3I; Aβ_42_, P < 0.001, Fig. 3J; T-tau, P < 0.001 Fig. 3K; P-tau, P < 0.001, Fig. 3L, Additional file 1: Table S1).

## Discussion

Here, we demonstrate that the molecular composition of ventricular CSF differs from that of the lumbar compartment in iNPH patients. A range of common neuropathologies, e.g., Alzheimer’s disease [7–9], multiple sclerosis [10], neuroborreliosis [11–13], and Creutzfeldt Jakob’s disease [14–16] relies on diagnostic CSF analysis during the clinical work-up. For this purpose, CSF is sampled from the lumbar region, for ethical reasons and technical ease, rather than from the ventricular compartment, in which the CSF is in direct contact with the brain tissue. The compartmentalized distribution of a range of biomarkers thus imposes a challenge to the field of CSF diagnostics.

Several recent studies have illustrated a CSF gradient of select biomarkers [46–50], which we here extend with mass spectrometry-based proteomic analysis of CSF obtained from the two compartments in the same individuals. This procedure became ethically acceptable by enrolling iNPH patients, who received a lumbar puncture during their diagnostic work-up and a subsequent CSF-diversion neurosurgical procedure allowing access to ventricular CSF. Of the 1231 proteins detected in the mass spectrometry-based analysis of these two compartmental CSF samples, 216 proteins were detected at equal levels in both compartments and 16 only in the ventricular compartment. 22 proteins were significantly more abundant (> twofold) in the ventricular compartment while four proteins were significantly more abundant (> twofold) in the lumbar compartment. The remainder of the CSF proteins were either dispersed with a tendency towards a compartmental preference but did not reach the significance threshold (> twofold change, P < 0.05 after Bonferroni correlation, see Material and Methods), or were below limit of detection in one of the compartments.

We here identified 1231 proteins which is less than detected in a previous study on the proteome of human CSF [51]. This difference could arise from our workflow containing no fractionation prior to LC–MS. Moreover, CSF studies by MS-based proteomics are challenging due to the high dynamic range of protein abundances, which combined with the limited dynamic range of MS instruments means that only the most to medium abundant proteins will be identified. Our methodology is optimized for a hightroughput and therefore the LC gradient is predefined and short.

To quantify the compartmentalized distribution of commonly employed clinical biomarkers, we employed standard ELISA-based quantification. The obtained levels of these CSF proteins aligned with earlier published reports on these biomarkers (S100B, NfL, Aβ_40_, Aβ_42_, T-tau, and P-tau [50, 52–55]). S100B is an astrocytic calcium-binding peptide that is released by necrotic or damaged cells. It has diagnostic and prognostic value as a biomarker in different CNS pathologies including traumatic brain injury, subarachnoid hemorrhage, cerebral inflammation, and in neurodegenerative diseases [41, 56, 57]. In the patient samples we detected S100B at significantly higher levels in ventricular CSF than in the lumbar CSF of the same patients. S100B was, in addition, detected in the mass spectrometry-based analysis, and presented with an identical compartmentalized distribution whether analyzed by ELISA or mass spectrometry, thus validating our experimental approach. NfL is a scaffolding protein of the neuronal cytoskeleton that is highly expressed in large caliber myelinated axons with a function in axonal structural support and growth. From here it leaks into CSF upon axonal injury and is thus employed as a general biomarker of neurodegeneration [58]. Curiously, NfL displayed the opposite distribution to that of S100B, with a favored compartmentalization in the lumbar compartment. In a study it was found that levels of NfL and tau were increased in acute and chronic inflammatory polyneuropathies suggesting that the source of NfL released into the CSF in these patients is likely damaged proximal nerve roots, which are surrounded by CSF in the subarachnoid space of the spinal cord [59]. This could point to NfL being a more sensitive marker of “lower” nerve injuries in the medulla, nerve roots and peripheral nerves than other biomarkers. Hence one should be careful when interpreting NfL in the lumbal CSF because the levels here could be affected by extracerebral neurodegeneration. In contrast, S100B and tau may represent/reflect a more cerebral neurodegeneration. Of the clinically employed Alzheimer’s disease biomarkers, two (Aβ_40_, Aβ_42_) were detected at higher levels in the lumbar compartment, whereas tau (T-tau and P-tau) was lower in lumbar than in ventricular samples from iNPH patients/human samples. Although the lumbar elevation of Aβ_42_ and NfL align with a previous study, the elevated ventricular concentration of tau, here observed, conflicts those earlier findings [55]. This discrepancy could be due to sampling methods of the ventricular CSF. Here, we collected the ventricular CSF during surgery where the drain is inserted into the ventricles through the parenchyma, which could result in damaged cells releasing tau into the brain fluid. In the previous study [55], ventricular CSF samples were collected through puncture of the shunt valves several months after placement. This would allow the levels of tau to return to “normal”/low values before sampling. This could also explain the elevated levels of S100B in the ventricular CSF. S100B is an astrocyte specific marker of brain damage and increases rapidly in both blood and CSF upon events like traumatic brain injury and infectious diseases. The mode of sampling could contaminate the CSF with S100B released from damaged cells from the parenchyma [60]. The same pattern is not found in the porcine CSF where the levels of S100B is higher in the lumbar CSF than in the cerebellomedullar cisterns. In contrast to the sample collection in the human patients, only the meninges were penetrated in the pigs to collect the CSF with no damage to the parenchyma. It could therefore be speculated that the mode of sample collection could contribute to the increase in S100B in the human ventricular CSF.

The craniospinal fluid dynamics are complex and incompletely understood and complex. In- and outflow of blood with the cardiac cycle is a driving force from the cranial compartment towards the spinal compartment in systole which is reversed in diastole. Flow dynamics thus contain flow patterns in both caudal and cranial direction [61]. Diffusion on the other hand is always directed from a compartment/location with higher molecular concentration to one with lower concentration, e.g., from the site of production to the site of sampling. In this study we collected CSF from iNPH patients. NPH is a disease characterized by enlarged ventricles and thus an abnormal volume of ventricular CSF. Magnetic resonance imaging (MRI) has demonstrated altered CSF flow in a group of these patients [62]. Further, the findings suggest that the CSF dynamics have an impact in the CSF biochemistry between the ventricular and lumbar compartment [62]. Due to the possible alterations in CSF flow the results of this study might not be applicable to other conditions. However, for ethical reasons it is not possible to collect ventricular CSF from healthy individuals or patients not suffering from neurological conditions.

Rats with congenital hydrocephalus (H-Tx) display changes of proteomic and metabolic CSF profile compared to normal rats [63]. Whether such changes are associated with iNPH (in human subjects) is unknown, but one could speculate that an alteration in CSF flow could influence the composition of the proteins found in both the ventricular CSF and lumbar CSF. With the obvious limitation of the sampling interval between the time of the lumbar and the ventricular sampling, we determined the compartmental difference of the six selected biomarkers as a function of interval between the sampling. The Alzheimer’s disease biomarkers did not reveal a time dependency of the interval between sampling, whereas the ventricular-lumbar differences of S100B and NfL became slightly more prominent with sampling interval. Notably, lumbar CSF concentrations of T-tau, P-tau and NfL increase in the months/years post-surgery [55], possibly simply due to disease progression or change in CSF flow due to the inserted shunt. CSF extracted without time delay from experimental pigs revealed that all biomarkers were slightly, but significantly, higher in the lumbar compartment compared to the ventricular CSF, albeit with much smaller differences than those observed in the human samples. This lack of distinct compartmentalized CSF biomarker levels could originate in the near-simultaneous sampling in the two CSF compartments, from an inherent difference in CSF dynamics from that of humans, and/or the horizontal anatomy of this animal. Gravity influences both blood- and CSF distribution between the intracranial compartment and extracranial spaces. This is highlighted in studies of animals with extreme gravitational challenges [64, 65]. In addition, removing the gravitational influence on human physiology during space flight strongly influences fluid and pressure distribution of the cardiovascular and CSF systems [66]. Being bipedal or quadruped could thus explain the differences between the porcine and human results. However, since the same protein gradients have been found between the cerebromedullary and the lumbar compartment in canines [19], gravity alone cannot explain this difference. Additionally, it could be speculated that during the lumbar sampling CSF from flow the intracranial space to the lumbar space could result in contamination of the lumbar CSF with ventricular CSF as CSF protein concentrations appear to depend on the volume of fluid withdrawn during the lumbar puncture [67]. However, the spinal CSF volume has been estimated to 80 ml [68] and in the present study we withdraw 40 ml thus consider ventricular CSF contamination unlikely.

Another limitation is the heterogeneity of the sampling of ventricular CSF. The CSF samples were collected upon shunt insertion (n = 39), ETV (n = 1), tap test through shunt chamber (n = 3) or during insertion of EVD (n = 3). The major limitation of this issue is that some samples have been collected through plastic tubing or syringes and others not. This could affect the results as some proteins, amyloid beta specifically, stick to plastic surfaces and thus would not be collected in the sample vials. However, of the 46 samples collected only one sample (ETV) was not in contact with plastic ware *en route* from the ventricles to the sample vials. Although sampling was performed during different clinical/surgical procedures, 43/46 (93%) were obtained before the effect of treatment (i.e., during diagnostic procedures or during the primary treatment procedure) and thus represent the pre-treatment period.

## Conclusion

In conclusion, our findings demonstrate that although the CSF presents with an overall protein gradient along the neuroaxis, each protein/biomarker distributes in a specific manner in these two CSF compartments. One therefore cannot directly extrapolate biomarker levels measured in lumbar samples to ventricular CSF without a known lumbar/cranial index for the molecule. However, future research and clinical work may benefit from the comprehensive list of protein distribution in these two CSF compartments.

## Supplementary Information

**Additional file 1: Figure S1.** Normality test for biomarkers measured in human and porcine CSF (both compartments) by immunoassays.** Figure S2.** Pearson’s correlation coefficients to investigate the influence of BMI (**A**) and the age of the patients (**B**) on biomarker levels. BMI: body mass index; LCSF: lumbar cerebrospinal fluid; VCSF: ventricular cerebrospinal fluid.** Figure S3.** No signs of blood contamination in CSF from one CSF compartment over the other. Visual assessment of data obtained by mass spectrometry. Data demonstrate that lumbar and ventricular samples contains similar levels of erythrocytes, platelets and coagulation factors. a.u.: arbitrary units; LCSF: lumbar cerebrospinal fluid; VCSF: ventricular cerebrospinalvæske.** Table S1.** Clinical biomarkers in porcine CSF. The table summarizes levels of S100B, NfL, Aß_40_, Aß_42_, T-tau and P-tau in porcine CSF measured by ELISAs.

**Additional file 2.** Complete list of proteins detected in lumbar and ventricular CSF samples from iNPH patients quantified with mass spectrometry and divided into ‘higher lumbar’, ‘higher ventricular’, ‘similar’ and ‘unclear’.

## Data Availability

Data are available upon reasonable request to the corresponding author.

## Abbreviations

Aβ_40/42_: Amyloid beta 40/42
CSF: Cerebrospinal fluid
NfL: Neurofilament light
LCSF: Lumbar cerebrospinal fluid
iNPH: Idiopathic normal pressure hydrocephalus
P-tau: Phosphorylated tau
T-tau: Total tau
VCSF: Ventricular cerebrospinal fluid
