# Supplementary material for: Differential proteomic profile of lumbar and ventricular cerebrospinal fluid
Source: Fluids Barriers CNS. 2023 Jan 21;20:6. doi: 10.1186/s12987-022-00405-0 (PMC9863210; doi:10.1186/s12987-022-00405-0)
Supplement: Supplementary file 1 — Additional file 1: Figure S1. Normality test for biomarkers measured in human and porcine CSF (both compartments) by immunoassays. Figure S2. Pearson’s correlation coefficients to investigate the influence of BMI (A) and the age of the patients (B) on biomarker levels. BMI: body mass index; LCSF: lumbar cerebrospinal fluid; VCSF: ventricular cerebrospinal fluid. Figure S3. No signs of blood contamination in CSF from one CSF compartment over the other. Visual assessment of data obtained by mass spectrometry. Data demonstrate that lumbar and ventricular samples contains similar levels of erythrocytes, platelets and coagulation factors. a.u.: arbitrary units; LCSF: lumbar cerebrospinal fluid; VCSF: ventricular cerebrospinalvæske. Table S1. Clinical biomarkers in porcine CSF. The table summarizes levels of S100B, NfL, Aß40, Aß42, T-tau and P-tau in porcine CSF measured by ELISAs. [file 12987_2022_405_MOESM1_ESM.pdf]

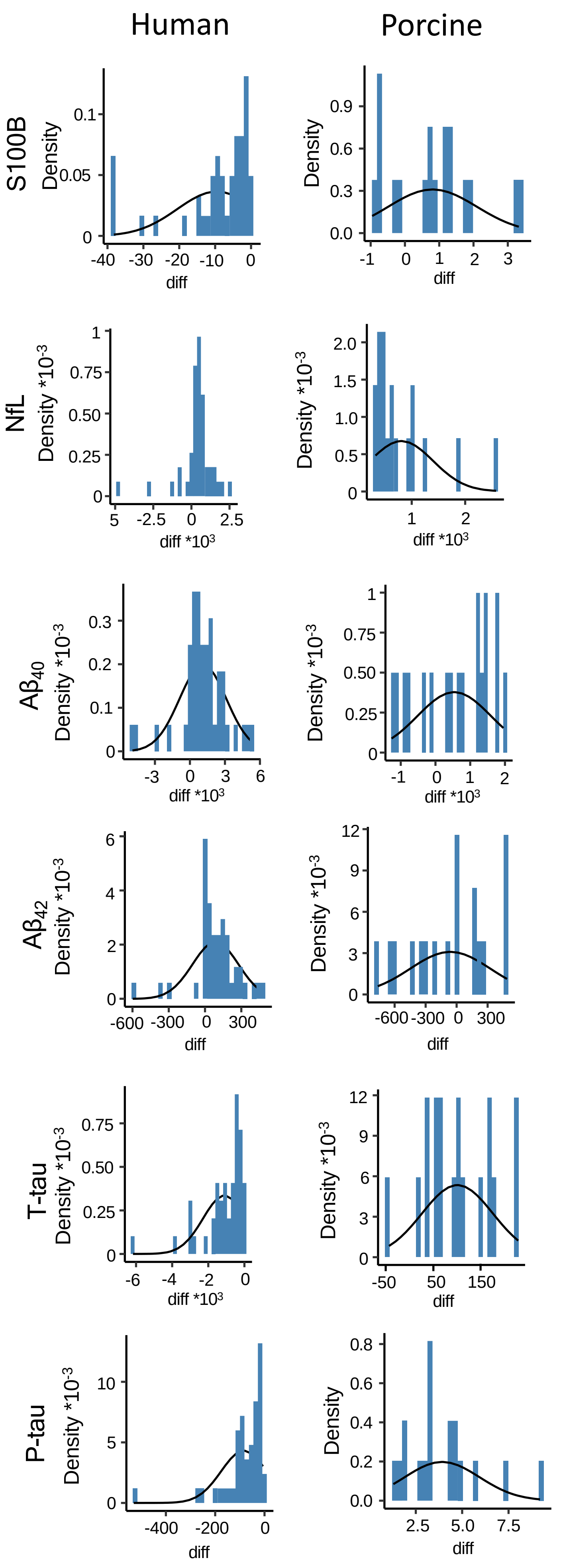

Supplementary figure S1. Normality test for biomarkers measured in human and porcine CSF (both compartments) by immunoassays.

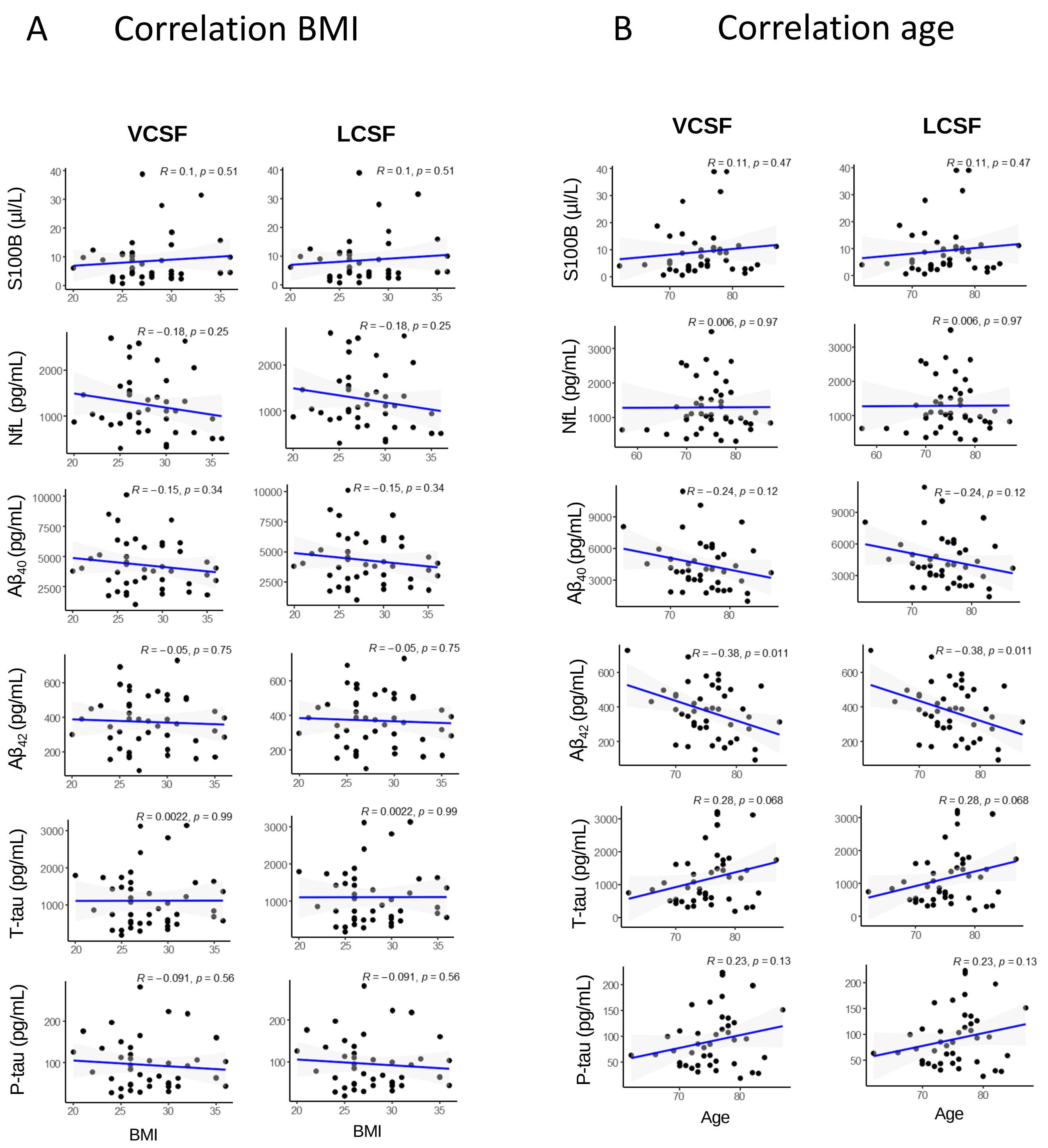

Supplementary figure S2. Pearson's correlation coefficients to investigate the influence of BMI **(A)** and the age of the patients **(B)** on biomarker levels. BMI: body mass index; LCSF: lumbar cerebrospinal fluid; VCSF: ventricular cerebrospinal fluid.

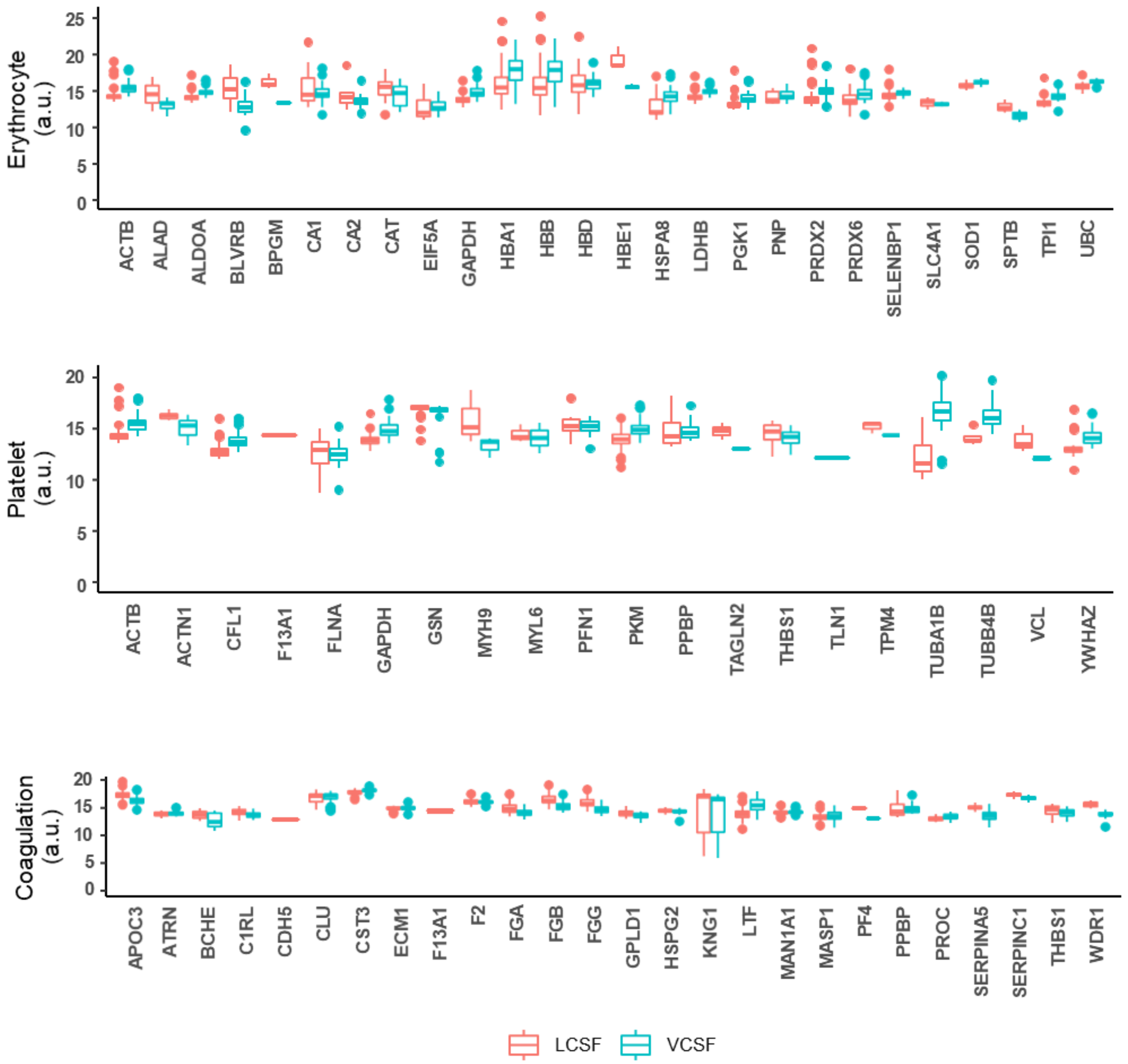

Supplementary figure S3. No signs of blood contamination in CSF from one CSF compartment over the other. Visual assessment of data obtained by mass spectrometry. Data demonstrate that lumbar and ventricular samples contains similar levels of erythrocytes, platelets and coagulation factors.
a.u.: arbitrary units; LCSF: lumbar cerebrospinal fluid; VCSF: ventricular cerebrospinal fluid

Porcine samples

Marker	No. of paired samples	LCSF		VCSF		P value
		Mean (min-max)	Std dev.	Mean (min-max)	Std dev.	
S100B (µg/L)	18	4.1 (1.7-8.2)	1.8	3.2 (2.1-6.3)	1.1	<0.05
NfL (pg/mL)	18	1252 (600-4070)	867	444 (160-1490)	383	<0.001
$A\beta_{40}$ (pg/mL)	18	5441 (3710-7604)	984	4904 (3533-6590)	720	<0.05
$A\beta_{42}$ (pg/mL)	18	826 (612-1050)	132	646 (458-812)	96	<0.001
T-tau (pg/mL)	18	384 (302-496)	57	284 (149-381)	62	<0.001
P-tau (pg/mL)	18	11.2 (7.8-17.5)	2.3	7.3 (4.6-9.3)	1.4	<0.001

Supplementary Table S1. Clinical biomarkers in porcine CSF. The table summarizes levels of S100B, NfL, $A\beta_{40}$, $A\beta_{42}$, T-tau and P-tau in porcine CSF measured by ELISAs.
