# Supplementary material for: Differential proteomic profile of lumbar and ventricular cerebrospinal fluid
Source: Fluids Barriers CNS. 2023 Jan 21;20:6. doi: 10.1186/s12987-022-00405-0 (PMC9863210; doi:10.1186/s12987-022-00405-0)
Supplement: Supplementary file 2 — Additional file 2. Complete list of proteins detected in lumbar and ventricular CSF samples from iNPH patients quantified with mass spectrometry and divided into ‘higher lumbar’, ‘higher ventricular’, ‘similar’ and ‘unclear’. [file 12987_2022_405_MOESM2_ESM.docx]

| **Group** | **Gene name** | **Uniprot ID** | **Lumbar**  mean (SD) [N] | **Ventricular**  mean (SD) [N] | ***p* value** | ***p* adjust** |
| --- | --- | --- | --- | --- | --- | --- |
| Higher lumbar | FGB | P02675 | 16.4 (0.9) [43] | 15.3 (0.8) [43] | <0.0001 | <0.0001 |
| Higher lumbar | FGG | P02679 | 15.8 (0.8) [43] | 14.7 (0.8) [43] | <0.0001 | <0.0001 |
| Higher lumbar | SERPINA5 | P05154 | 15 (0.4) [43] | 13.4 (0.9) [43] | <0.0001 | <0.0001 |
| Higher lumbar | LIAS | A0A1W2PNQ5 | 15.6 (0.8) [41] | 14.4 (0.8) [31] | <0.0001 | <0.0001 |
| Higher ventricular | ACTB | P60709 | 14.5 (1) [43] | 15.5 (0.8) [43] | <0.0001 | 0.0007 |
| Higher ventricular | ACTBL2 | Q562R1 | 16.3 (1.1) [43] | 17.3 (0.9) [43] | <0.0001 | 0.0043 |
| Higher ventricular | ECRG4 | B8ZZE5 | 15 (0.7) [43] | 16.1 (1) [43] | <0.0001 | <0.0001 |
| Higher ventricular | F5 | A0A0A0MRJ7 | 14.4 (0.4) [43] | 15.4 (0.6) [43] | <0.0001 | <0.0001 |
| Higher ventricular | IGFBP7 | Q16270 | 16 (0.4) [43] | 17.3 (0.7) [43] | <0.0001 | <0.0001 |
| Higher ventricular | PPIA | P62937 | 14 (0.9) [43] | 15.3 (0.8) [43] | <0.0001 | <0.0001 |
| Higher ventricular | SPARC | P09486 | 15.3 (0.5) [43] | 16.7 (0.7) [43] | <0.0001 | <0.0001 |
| Higher ventricular | SPP1 | P10451-5 | 16.8 (0.9) [42] | 17.8 (0.7) [43] | <0.0001 | <0.0001 |
| Higher ventricular | YWHAZ | P63104 | 13.1 (0.9) [41] | 14.2 (0.8) [43] | <0.0001 | <0.0001 |
| Higher ventricular | S100B | P04271 | 12.6 (0.6) [39] | 15.5 (1.4) [42] | <0.0001 | <0.0001 |
| Higher ventricular |  | E9PNW4 | 14.2 (1.4) [37] | 15.2 (0.9) [43] | <0.0001 | 0.0049 |
| Higher ventricular | SIAE | Q9HAT2 | 13.9 (0.6) [37] | 14.9 (0.7) [41] | <0.0001 | <0.0001 |
| Higher ventricular | CBR1 | P16152 | 13.1 (0.6) [34] | 14.3 (1) [42] | <0.0001 | <0.0001 |
| Higher ventricular | CFL1 | E9PK25 | 12.9 (0.8) [33] | 13.9 (0.8) [41] | <0.0001 | 0.0023 |
| Higher ventricular | ENO2 | P09104 | 13.6 (0.9) [29] | 14.8 (0.8) [43] | <0.0001 | 0.0004 |
| Higher ventricular | GAPDH | P04406 | 13.9 (0.7) [29] | 14.9 (0.9) [43] | <0.0001 | 0.0002 |
| Higher ventricular | YWHAE | P62258 | 12.4 (1.2) [26] | 13.8 (1) [43] | <0.0001 | 0.0017 |
| Higher ventricular | LTF | E7EQB2 | 13.7 (1.2) [31] | 15.3 (1.3) [37] | <0.0001 | 0.0009 |
| Higher ventricular | ENO1 | P06733 | 13.8 (1.3) [24] | 15.2 (0.9) [42] | <0.0001 | 0.0177 |
| Higher ventricular | PKM | P14618-2 | 13.9 (1) [29] | 15.1 (1) [37] | <0.0001 | 0.0102 |
| Higher ventricular | NDRG2 | Q9UN36-2 | 12.9 (1.1) [19] | 13.9 (0.8) [38] | <0.0001 | 0.012 |
| Higher ventricular | QDPR | P09417 | 13.8 (1) [14] | 14.8 (0.9) [40] | 0.0001 | 0.041 |
| Similar | AGT | P01019 | 17 (0.3) [43] | 17 (0.4) [43] | 0.0538 | 1 |
| Similar | APOH | P02749 | 15.6 (0.4) [43] | 15.5 (0.3) [43] | 0.2066 | 1 |
| Similar | C1QC | P02747 | 16.8 (0.4) [43] | 16.8 (0.3) [43] | 0.3033 | 1 |
| Similar | C1R | B4DPQ0 | 15.5 (0.3) [43] | 15.6 (0.3) [43] | 0.2429 | 1 |
| Similar | C1S | P09871 | 16.2 (0.2) [43] | 16.2 (0.3) [43] | 0.5856 | 1 |
| Similar | C4A | A0A0G2JPR0 | 15.4 (0.8) [43] | 15.5 (0.7) [43] | 0.3446 | 1 |
| Similar | C4B | P0C0L5 | 16.7 (0.3) [43] | 16.7 (0.3) [43] | 0.9364 | 1 |
| Similar | C8A | P07357 | 15.3 (0.5) [43] | 15.2 (0.5) [43] | 0.085 | 1 |
| Similar | CD14 | P08571 | 16.5 (0.4) [43] | 16.4 (0.4) [43] | 0.2281 | 1 |
| Similar | CFHR1 | B1AKG0 | 14.9 (0.6) [43] | 14.8 (0.7) [43] | 0.5021 | 1 |
| Similar | CLEC3B | E9PHK0 | 16.9 (0.5) [43] | 16.9 (0.3) [43] | 0.4987 | 1 |
| Similar | CLU | P10909 | 17.3 (0.3) [43] | 17.3 (0.3) [43] | 0.3623 | 1 |
| Similar | COL18A1 | P39060 | 15.2 (0.4) [43] | 15.2 (0.3) [43] | 0.5888 | 1 |
| Similar | COL6A3 | P12111 | 13.9 (0.4) [43] | 13.7 (0.5) [43] | 0.0555 | 1 |
| Similar | ECM1 | Q16610 | 14.8 (0.3) [43] | 14.9 (0.4) [43] | 0.5068 | 1 |
| Similar | F2 | P00734 | 16 (0.5) [43] | 15.9 (0.3) [43] | 0.4207 | 1 |
| Similar | FN1 | P02751 | 15.9 (0.3) [43] | 15.9 (0.3) [43] | 0.5461 | 1 |
| Similar | FSTL1 | Q12841 | 13.4 (0.9) [43] | 13.6 (0.6) [43] | 0.2688 | 1 |
| Similar | HP | P00738 | 17.4 (1.2) [43] | 17.1 (1.1) [43] | 0.1022 | 1 |
| Similar | HPR | P00739 | 14.7 (1) [43] | 14.5 (1) [43] | 0.1184 | 1 |
| Similar | HPX | P02790 | 18.2 (0.5) [43] | 18.1 (0.3) [43] | 0.2598 | 1 |
| Similar | IGFBP2 | P18065 | 14.1 (0.5) [43] | 14.1 (0.6) [43] | 0.6867 | 1 |
| Similar | IGKV3-15 | P01624 | 17.5 (0.5) [43] | 17.4 (0.6) [43] | 0.3142 | 1 |
| Similar | IGLV1-47 | P01700 | 16.3 (0.8) [43] | 16 (0.6) [43] | 0.0664 | 1 |
| Similar | LYVE1 | Q9Y5Y7 | 14.6 (0.7) [43] | 14.5 (0.5) [43] | 0.5848 | 1 |
| Similar | LYZ | A0A0B4J259 | 16.8 (0.6) [43] | 16.8 (0.5) [43] | 0.6071 | 1 |
| Similar | MGP | P08493 | 15.6 (0.4) [43] | 15.5 (0.5) [43] | 0.1192 | 1 |
| Similar | PI16 | Q6UXB8 | 13.8 (0.6) [43] | 13.7 (0.6) [43] | 0.3942 | 1 |
| Similar | PLTP | P55058 | 16 (0.3) [43] | 16 (0.3) [43] | 0.5546 | 1 |
| Similar | PROCR | Q9UNN8 | 14.2 (0.8) [43] | 14.3 (0.8) [43] | 0.2086 | 1 |
| Similar | RBP4 | P02753 | 16.3 (0.3) [43] | 16.4 (0.4) [43] | 0.2881 | 1 |
| Similar | RNASE1 | P07998 | 12.4 (0.8) [43] | 12.4 (0.6) [43] | 0.7251 | 1 |
| Similar | SCRG1 | O75711 | 16.9 (0.4) [43] | 17.1 (0.7) [43] | 0.059 | 1 |
| Similar | SELL | P14151 | 15.2 (0.4) [43] | 15.1 (0.5) [43] | 0.057 | 1 |
| Similar | TAGLN | Q01995 | 14.1 (0.6) [43] | 14 (0.7) [43] | 0.4089 | 1 |
| Similar | TF | P02787 | 17.9 (0.3) [43] | 17.9 (0.2) [43] | 0.7373 | 1 |
| Similar | TGFBI | Q15582 | 14.5 (0.6) [43] | 14.4 (0.3) [43] | 0.3039 | 1 |
| Similar | APOB | P04114 | 13.5 (1.2) [42] | 13.7 (1.1) [43] | 0.4252 | 1 |
| Similar | C3 | M0R1Q1 | 17.2 (0.6) [42] | 17.1 (0.6) [43] | 0.5144 | 1 |
| Similar | CADM3 | Q8N126 | 15.7 (0.7) [42] | 15.7 (0.5) [43] | 0.8128 | 1 |
| Similar | CHI3L2 | Q15782 | 13.2 (0.8) [43] | 13.1 (0.7) [42] | 0.6943 | 1 |
| Similar | FCGBP | Q9Y6R7 | 14.3 (0.8) [42] | 14.3 (0.6) [43] | 0.5064 | 1 |
| Similar | FETUB | Q9UGM5 | 14.2 (0.9) [42] | 14 (0.6) [43] | 0.1134 | 1 |
| Similar | LCAT | P04180 | 15 (0.7) [43] | 15 (0.6) [42] | 0.853 | 1 |
| Similar | VSIG4 | Q9Y279 | 13.1 (1.1) [42] | 13.1 (0.5) [43] | 0.9567 | 1 |
| Similar | C16orf89 | A0A0A0MT71 | 14.5 (0.5) [41] | 14.6 (0.4) [43] | 0.1869 | 1 |
| Similar | DCN | P07585 | 14 (0.8) [42] | 14.1 (0.8) [42] | 0.7366 | 1 |
| Similar | ECM2 | O94769 | 14.8 (0.6) [41] | 14.7 (0.6) [43] | 0.3613 | 1 |
| Similar | IGHV1OR15-1 | A0A075B7D0 | 17 (1.3) [42] | 16.9 (1.2) [42] | 0.2885 | 1 |
| Similar | MAN1A1 | P33908 | 14.1 (0.4) [42] | 14.2 (0.3) [42] | 0.0772 | 1 |
| Similar | AEBP1 | Q8IUX7 | 13.9 (0.5) [40] | 13.8 (0.5) [43] | 0.2336 | 1 |
| Similar | CD99 | P14209 | 16.2 (0.5) [40] | 16.4 (0.7) [43] | 0.1103 | 1 |
| Similar | CLSTN3 | Q9BQT9 | 12.7 (2) [40] | 12.7 (1.8) [43] | 0.4641 | 1 |
| Similar | CNTNAP4 | A0A087WTA1 | 14.3 (0.7) [40] | 14.1 (0.7) [43] | 0.1463 | 1 |
| Similar | FBLN5 | G3V4U0 | 15.3 (0.8) [40] | 15.1 (0.7) [43] | 0.2339 | 1 |
| Similar | IDS | P22304 | 14.7 (0.7) [40] | 14.7 (0.6) [43] | 0.7741 | 1 |
| Similar | ITIH5 | C9J2H1 | 13.8 (0.4) [40] | 13.8 (0.3) [43] | 0.5623 | 1 |
| Similar | MFAP4 | K7ES70 | 14.4 (0.5) [40] | 14.5 (0.6) [43] | 0.2408 | 1 |
| Similar | NRXN2 | G5E9G7 | 15 (0.6) [40] | 15 (0.6) [43] | 0.3998 | 1 |
| Similar | QPCT | Q16769 | 13.8 (0.6) [40] | 14 (0.6) [43] | 0.1671 | 1 |
| Similar | SELENOP | A0A182DWH7 | 15.5 (0.7) [41] | 15.5 (0.6) [42] | 0.8339 | 1 |
| Similar | SLITRK4 | Q8IW52 | 13.4 (0.5) [40] | 13.5 (0.5) [43] | 0.225 | 1 |
| Similar | TCN2 | B5MBX2 | 15.3 (1.3) [41] | 15.5 (1.3) [42] | 0.4625 | 1 |
| Similar | TIMP1 | P01033 | 16.8 (1.2) [43] | 16.6 (0.4) [40] | 0.1759 | 1 |
| Similar | TREM2 | Q9NZC2 | 14 (0.9) [43] | 13.8 (0.9) [40] | 0.1183 | 1 |
| Similar | PPIC | P45877 | 14.6 (0.4) [41] | 14.8 (0.4) [41] | 0.23 | 1 |
| Similar | ADAM22 | F8WAD8 | 12.3 (1.4) [38] | 12.7 (1.9) [43] | 0.2906 | 1 |
| Similar | IGKV1-17 | P01599 | 15.4 (0.7) [41] | 15.3 (0.7) [40] | 0.174 | 1 |
| Similar | MYH11 | P35749 | 15.7 (1.6) [39] | 15.8 (1) [42] | 0.921 | 1 |
| Similar | NPY | P01303 | 14 (1.2) [38] | 14.4 (1.2) [43] | 0.2607 | 1 |
| Similar | ROBO1 | Q9Y6N7 | 14.1 (0.4) [39] | 14.1 (0.5) [42] | 0.6441 | 1 |
| Similar | CDH4 | P55283 | 13.2 (0.5) [37] | 13.3 (0.7) [43] | 0.1162 | 1 |
| Similar | IGHV3-64D | A0A0J9YX35 | 15.9 (0.6) [38] | 15.8 (0.7) [42] | 0.95 | 1 |
| Similar | VSTM2B | A6NLU5 | 13.9 (0.9) [38] | 14.1 (1) [42] | 0.2159 | 1 |
| Similar | DNER | Q8NFT8 | 12.1 (0.4) [39] | 12.1 (0.5) [40] | 0.2416 | 1 |
| Similar | NID2 | Q14112 | 14.1 (0.5) [39] | 13.9 (0.5) [40] | 0.2568 | 1 |
| Similar | NRP1 | E7EX60 | 13 (0.5) [37] | 12.9 (0.5) [42] | 0.4418 | 1 |
| Similar | ADGRL1 | O94910 | 13.9 (0.7) [36] | 13.9 (0.6) [42] | 0.9002 | 1 |
| Similar | AGA | P20933 | 13.9 (1.6) [35] | 14 (1.8) [43] | 0.8066 | 1 |
| Similar | GANAB | Q14697 | 12.5 (0.6) [37] | 12.7 (0.6) [41] | 0.1182 | 1 |
| Similar | IGKV3-7 | A0A075B6H7 | 18.6 (1.2) [38] | 18.4 (1.1) [40] | 0.5379 | 1 |
| Similar | LY6H | O94772 | 11.7 (0.8) [38] | 11.8 (0.9) [40] | 0.6356 | 1 |
| Similar | PTPRN2 | Q92932 | 15.4 (0.8) [35] | 15.5 (0.6) [43] | 0.8483 | 1 |
| Similar | RELN | J3KQ66 | 14.6 (0.5) [39] | 14.7 (0.6) [39] | 0.253 | 1 |
| Similar | HSPA5 | P11021 | 14.1 (0.7) [34] | 14.3 (0.2) [43] | 0.0888 | 1 |
| Similar | BGN | P21810 | 13.2 (0.8) [35] | 13.6 (1) [41] | 0.0798 | 1 |
| Similar | IGLV3-19 | P01714 | 14.5 (0.6) [41] | 14.2 (0.8) [35] | 0.0583 | 1 |
| Similar | ITIH3 | Q06033 | 13.6 (0.9) [42] | 13.5 (0.8) [34] | 0.2399 | 1 |
| Similar | MGAT1 | P26572 | 14 (0.5) [38] | 14.2 (0.9) [38] | 0.2206 | 1 |
| Similar | VIM | P08670 | 13.2 (1.4) [42] | 12.8 (1.2) [34] | 0.2226 | 1 |
| Similar | L1CAM | P32004 | 12.6 (0.6) [33] | 12.6 (0.7) [42] | 0.1582 | 1 |
| Similar | SLC3A2 | F5GZS6 | 13.1 (0.6) [35] | 13 (0.4) [40] | 0.3919 | 1 |
| Similar | EFCAB14 | O75071 | 13.9 (0.4) [37] | 14 (0.6) [37] | 0.0797 | 1 |
| Similar | LTBP2 | G3V3X5 | 12.6 (0.7) [34] | 12.5 (0.6) [40] | 0.2346 | 1 |
| Similar | OLFM1 | Q99784 | 14.1 (0.4) [33] | 14.1 (0.4) [41] | 0.474 | 1 |
| Similar | TIMP2 | P16035 | 15.8 (0.3) [31] | 15.9 (0.4) [42] | 0.6728 | 1 |
| Similar | COL15A1 | A0A087X0K0 | 13.8 (0.6) [41] | 13.9 (0.5) [31] | 0.4347 | 1 |
| Similar | CTSB | P07858 | 13.5 (1.1) [31] | 13.7 (0.6) [41] | 0.2765 | 1 |
| Similar |  | A0A0G2JRQ6 | 16 (0.8) [35] | 15.6 (0.7) [37] | 0.2591 | 1 |
| Similar | B3GALNT1 | O75752 | 13.3 (0.7) [30] | 13.4 (0.6) [41] | 0.616 | 1 |
| Similar | LBP | P18428 | 13.5 (0.7) [35] | 13.5 (0.5) [36] | 0.6888 | 1 |
| Similar | MRC1 | P22897 | 13.8 (1.3) [35] | 13.6 (0.7) [36] | 0.5479 | 1 |
| Similar | CLEC11A | Q9Y240 | 13.3 (0.5) [36] | 13.2 (0.7) [34] | 0.4072 | 1 |
| Similar | TWSG1 | J3QS03 | 14.6 (0.3) [32] | 14.6 (0.2) [38] | 0.0578 | 1 |
| Similar | C8G | P07360 | 14.2 (0.9) [31] | 14.3 (0.7) [38] | 0.7059 | 1 |
| Similar | FBLN7 | Q53RD9 | 12.5 (0.5) [31] | 12.6 (0.4) [38] | 0.0994 | 1 |
| Similar | LAMP1 | P11279 | 12.9 (0.4) [31] | 13 (0.5) [38] | 0.0908 | 1 |
| Similar | KRT2 | P35908 | 14.3 (1.6) [31] | 14.7 (1.7) [37] | 0.1795 | 1 |
| Similar | PCDHAC2 | Q9Y5I4 | 13.2 (1.1) [33] | 13.4 (0.9) [35] | 0.5925 | 1 |
| Similar | LAMA2 | A0A087WX80 | 11.2 (1.3) [37] | 11 (1.3) [30] | 0.2779 | 1 |
| Similar | PDGFB | A9UJN9 | 13.7 (0.8) [34] | 14.2 (0.8) [33] | 0.2599 | 1 |
| Similar | SEMA3G | Q9NS98 | 12.9 (0.7) [35] | 13.1 (0.6) [32] | 0.3457 | 1 |
| Similar | ADAMTS4 | O75173 | 14.4 (0.3) [27] | 14.6 (0.4) [39] | 0.0548 | 1 |
| Similar | B2M | H0YLF3 | 12.8 (0.4) [30] | 13.1 (0.4) [36] | 0.0686 | 1 |
| Similar | HSP90B1 | P14625 | 13.7 (0.9) [29] | 13.8 (0.4) [37] | 0.3671 | 1 |
| Similar | PAPLN | O95428 | 13.2 (0.4) [38] | 13.3 (0.4) [28] | 0.8118 | 1 |
| Similar | SLITRK1 | Q96PX8 | 13.4 (0.5) [33] | 13.4 (0.6) [33] | 0.771 | 1 |
| Similar | EFNA1 | P20827 | 13.3 (0.9) [28] | 13.5 (1.2) [37] | 0.8261 | 1 |
| Similar | SDF4 | Q9BRK5 | 13.1 (1.1) [28] | 13.5 (0.8) [37] | 0.1011 | 1 |
| Similar | CASP14 | P31944 | 14.2 (1.1) [39] | 14.1 (1) [25] | 0.3461 | 1 |
| Similar | GDA | Q9Y2T3 | 13.4 (0.7) [21] | 13.7 (0.7) [43] | 0.1542 | 1 |
| Similar | LAMC1 | P11047 | 14.6 (2.3) [32] | 14.1 (1.1) [32] | 0.2049 | 1 |
| Similar | CALY | Q9NYX4 | 13.8 (0.8) [22] | 13.6 (0.7) [41] | 0.2571 | 1 |
| Similar | MASP1 | P48740-2 | 13.1 (0.7) [26] | 13.5 (1) [37] | 0.2572 | 1 |
| Similar | PLD4 | F5H2B5 | 13.5 (0.5) [27] | 13.7 (0.6) [36] | 0.0567 | 1 |
| Similar | SPOCK2 | Q92563 | 14 (0.5) [23] | 13.9 (0.7) [39] | 0.8418 | 1 |
| Similar | TALDO1 | P37837 | 13.6 (1.9) [23] | 13.8 (1) [39] | 0.4742 | 1 |
| Similar | ADAMTS1 | Q9UHI8 | 14.5 (0.3) [27] | 14.6 (0.3) [34] | 0.1312 | 1 |
| Similar | CCP110 | O43303 | 21.7 (0.7) [29] | 21.6 (0.8) [32] | 0.7766 | 1 |
| Similar | CD163 | C9JHR8 | 13.7 (1.1) [29] | 13.5 (0.6) [32] | 0.6469 | 1 |
| Similar | RNASE6 | Q93091 | 13.9 (0.6) [23] | 13.7 (0.7) [38] | 0.1578 | 1 |
| Similar | GAP43 | P17677 | 11.4 (1.1) [23] | 11.6 (0.8) [37] | 0.2205 | 1 |
| Similar | PDIA3 | P30101 | 13.5 (0.9) [23] | 13.5 (0.6) [37] | 0.5812 | 1 |
| Similar | PCDH17 | O14917 | 12 (0.5) [27] | 11.8 (0.8) [32] | 0.2609 | 1 |
| Similar | B3GNT2 | Q9NY97 | 12.2 (1) [24] | 12.3 (1) [34] | 0.7739 | 1 |
| Similar | C1QTNF5 | Q9BXJ0 | 12.7 (0.8) [16] | 13.2 (0.7) [42] | 0.1176 | 1 |
| Similar | COL3A1 | P02461 | 13.6 (0.9) [28] | 13.2 (0.7) [30] | 0.2511 | 1 |
| Similar | SLC39A10 | Q9ULF5 | 12.6 (0.4) [26] | 12.5 (0.6) [32] | 0.9907 | 1 |
| Similar | CHST10 | O43529 | 17.6 (0.5) [24] | 18.2 (1.8) [33] | 0.4091 | 1 |
| Similar | DDR1 | A0A0A0MSX3 | 13.8 (1.1) [20] | 14 (0.5) [36] | 0.2029 | 1 |
| Similar | KRT10 | P13645 | 13.3 (1.4) [24] | 13.9 (1.3) [32] | 0.0728 | 1 |
| Similar | MATN2 | O00339 | 12.9 (0.3) [32] | 12.7 (0.5) [24] | 0.0937 | 1 |
| Similar | PLOD1 | Q02809 | 12.7 (0.4) [21] | 12.9 (0.6) [35] | 0.751 | 1 |
| Similar | CA1 | P00915 | 15.5 (2.4) [20] | 14.8 (1.3) [35] | 0.1499 | 1 |
| Similar | CTSC | P53634 | 13.2 (0.6) [30] | 13 (0.5) [24] | 0.0537 | 1 |
| Similar | DCC | E7EQM8 | 13.4 (0.4) [23] | 13.4 (0.5) [31] | 0.8465 | 1 |
| Similar | F10 | P00742 | 14.3 (1) [24] | 14.2 (0.4) [30] | 0.6164 | 1 |
| Similar | FTL | P02792 | 13.7 (2.2) [16] | 13.2 (0.5) [38] | 0.471 | 1 |
| Similar | LAMB2 | P55268 | 12.3 (0.3) [22] | 12.3 (0.5) [32] | 0.5633 | 1 |
| Similar | LGALS1 | P09382 | 14.1 (0.8) [20] | 14.5 (0.5) [34] | 0.1419 | 1 |
| Similar | C4BPA | P04003 | 12.6 (1.5) [28] | 12.3 (1.2) [25] | 0.3114 | 1 |
| Similar | FRRS1L | Q9P0K9 | 10.4 (2.3) [20] | 10.5 (2.1) [33] | 0.1674 | 1 |
| Similar | GOLIM4 | F8W785 | 10.9 (1.6) [26] | 10.1 (1.5) [27] | 0.1602 | 1 |
| Similar | LCP1 | P13796 | 14 (1) [27] | 13.8 (0.5) [26] | 0.3594 | 1 |
| Similar | GPC1 | P35052 | 12.8 (2.2) [20] | 12.9 (1.8) [32] | 0.8257 | 1 |
| Similar | HBD | P02042 | 16.3 (2.5) [16] | 16 (0.9) [36] | 0.6806 | 1 |
| Similar | PCDH7 | O60245 | 12.4 (0.4) [23] | 12.6 (0.5) [29] | 0.1241 | 1 |
| Similar | RP1 | P56715 | 15.1 (0.6) [27] | 14.8 (0.7) [25] | 0.0523 | 1 |
| Similar | IGHV3OR16-12 | A0A075B7B8 | 13.2 (0.5) [28] | 13 (0.4) [23] | 0.1588 | 1 |
| Similar | KRT6A | P02538 | 12.7 (1.1) [21] | 13.5 (2) [30] | 0.3138 | 1 |
| Similar | KRT77 | Q7Z794 | 14.9 (1.2) [20] | 15.1 (1.7) [31] | 0.8446 | 1 |
| Similar | ANG | P03950 | 13.8 (0.4) [20] | 13.9 (0.6) [30] | 0.2679 | 1 |
| Similar | AXL | P30530 | 15 (0.6) [17] | 15.1 (1) [33] | 0.6979 | 1 |
| Similar | IGKV1-37 | A0A075B6S9 | 16.6 (1.2) [27] | 15.8 (0.9) [23] | 0.0567 | 1 |
| Similar | KRT9 | P35527 | 14.1 (1.1) [20] | 14.2 (1.4) [30] | 0.8826 | 1 |
| Similar | MASP1 | P48740 | 13.4 (0.7) [17] | 13.7 (0.9) [33] | 0.5679 | 1 |
| Similar | PPT1 | A0A286YFE3 | 12.3 (0.4) [15] | 12.5 (0.5) [35] | 0.1027 | 1 |
| Similar | RTN4R | H7C0V4 | 13.9 (1) [18] | 13.6 (0.6) [32] | 0.9247 | 1 |
| Similar | CSPG4 | Q6UVK1 | 11.5 (0.8) [29] | 11.4 (0.8) [20] | 0.9147 | 1 |
| Similar | NOMO1 | A0A087X117 | 13.2 (2.4) [24] | 14.1 (2) [25] | 0.9261 | 1 |
| Similar | NTRK2 | Q16620 | 15.1 (0.5) [15] | 15.2 (0.5) [34] | 0.1315 | 1 |
| Similar | OLFML3 | B4DNG0 | 12.5 (1) [25] | 12.6 (0.8) [24] | 0.1166 | 1 |
| Similar | SELENOM | A0A087WWF1 | 12.9 (0.6) [23] | 13.2 (0.6) [26] | 0.3201 | 1 |
| Similar | ST6GALNAC1 | Q9NSC7 | 13 (0.5) [26] | 13.1 (0.5) [23] | 0.736 | 1 |
| Similar | MINPP1 | Q9UNW1 | 14.6 (1.3) [24] | 14.4 (1.3) [24] | 0.8981 | 1 |
| Similar | OPCML | Q14982 | 14.5 (0.8) [21] | 14.4 (1.1) [27] | 0.8492 | 1 |
| Similar | SLITRK5 | O94991 | 12.4 (1.4) [23] | 13.2 (1.2) [25] | 0.1411 | 1 |
| Similar | THBS2 | P35442 | 13.8 (0.5) [21] | 13.9 (0.5) [27] | 0.801 | 1 |
| Similar | CD109 | Q6YHK3 | 12.2 (1.4) [16] | 12.1 (0.7) [31] | 0.8543 | 1 |
| Similar | FCGR2A | P12318 | 14.2 (0.3) [19] | 14.3 (0.4) [28] | 0.6389 | 1 |
| Similar | GLOD4 | F6TLX2 | 15.7 (1.1) [20] | 15.3 (1.4) [27] | 0.3396 | 1 |
| Similar | HSPA13 | P48723 | 12.9 (0.5) [19] | 13 (0.9) [28] | 0.7618 | 1 |
| Similar | SERPINA10 | G3V2W1 | 11.7 (0.8) [30] | 11.7 (1.1) [17] | 0.3202 | 1 |
| Similar | CSTB | P04080 | 14.2 (1.3) [14] | 14.2 (0.6) [32] | 0.947 | 1 |
| Similar | IGHD | A0A0A0MS09 | 14.5 (0.9) [23] | 14.2 (1.1) [23] | 0.0916 | 1 |
| Similar | NCAM1 | H7BYX6 | 12.1 (1.3) [19] | 11.9 (1.2) [27] | 0.4727 | 1 |
| Similar | PFN1 | P07737 | 15.3 (1.1) [14] | 15.2 (0.7) [32] | 0.9991 | 1 |
| Similar | QSOX2 | Q6ZRP7 | 10.8 (0.7) [23] | 10.7 (0.7) [23] | 0.2824 | 1 |
| Similar | TPT1 | A0A0B4J2C3 | 16.9 (0.5) [20] | 16.8 (0.5) [26] | 0.1601 | 1 |
| Similar | HS6ST3 | Q8IZP7 | 14.2 (1.4) [17] | 13.9 (1.5) [28] | 0.5068 | 1 |
| Similar | IGKV1-16 | P04430 | 14.6 (0.6) [26] | 14.3 (0.7) [19] | 0.0504 | 1 |
| Similar | PDGFA | A0A0A0MSC4 | 15.4 (0.6) [17] | 15.6 (0.4) [28] | 0.7798 | 1 |
| Similar | PVALB | B8ZZ19 | 13.3 (0.6) [20] | 13.4 (0.7) [24] | 0.1167 | 1 |
| Similar | COCH | O43405 | 13.7 (0.4) [12] | 13.9 (0.6) [30] | 0.1067 | 1 |
| Similar | CXCL16 | Q9H2A7 | 14.5 (0.2) [15] | 14.5 (0.3) [27] | 0.6102 | 1 |
| Similar | GALNS | P34059 | 13.6 (2.1) [19] | 13.3 (2.1) [23] | 0.687 | 1 |
| Similar | CLU | P10909-3 | 15.7 (0.9) [25] | 15.6 (1) [16] | 0.8192 | 1 |
| Similar | COTL1 | Q14019 | 12.5 (0.9) [13] | 12.7 (0.6) [28] | 0.3936 | 1 |
| Similar | GAA | P10253 | 12.8 (0.7) [10] | 13.1 (1.3) [31] | 0.0906 | 1 |
| Similar | NPTX2 | P47972 | 13.3 (1) [16] | 13.4 (0.8) [25] | 0.7576 | 1 |
| Similar | CELSR2 | Q9HCU4 | 13.9 (1.1) [11] | 13.9 (0.6) [29] | 0.5178 | 1 |
| Similar | IGHV2-5 | P01817 | 12.1 (1.2) [23] | 12.5 (1.3) [17] | 0.3333 | 1 |
| Similar | IGKV1-39 | P01597 | 19.7 (0.9) [19] | 19.9 (0.7) [21] | 0.5816 | 1 |
| Similar | CST6 | Q15828 | 13.3 (0.4) [18] | 13.3 (0.5) [21] | 0.6519 | 1 |
| Similar | ADAM11 | B4DKD2 | 13.2 (0.4) [12] | 13 (0.4) [26] | 0.4644 | 1 |
| Similar | CALB1 | P05937 | 13.2 (0.7) [15] | 13.5 (1.2) [23] | 0.3585 | 1 |
| Similar | CCL14 | A0A087X089 | 13.7 (0.4) [16] | 13.7 (0.5) [22] | 0.3565 | 1 |
| Similar | MT3 | H3BPK2 | 8.8 (0.6) [16] | 10 (2.4) [22] | 0.5952 | 1 |
| Similar | IGHV1-18 | A0A0C4DH31 | 14.3 (0.8) [18] | 14.3 (0.5) [19] | 0.6146 | 1 |
| Similar | NSF | I3L0N3 | 12.9 (0.4) [14] | 12.9 (1.1) [23] | 0.4842 | 1 |
| Similar | DSC1 | Q08554 | 14.8 (0.4) [16] | 14.8 (0.3) [18] | 0.8681 | 1 |
| Similar | MAN2B2 | E9PCD7 | 13.4 (1.5) [17] | 14.2 (1.4) [15] | 0.6471 | 1 |
| Similar | B3GNT9 | Q6UX72 | 7.6 (1) [18] | 6.9 (0.8) [13] | 0.1501 | 1 |
| Similar | CDH15 | P55291 | 12.5 (0.9) [12] | 12.2 (1.1) [18] | 0.7892 | 1 |
| Similar | IGHV4-28 | A0A0C4DH34 | 9.7 (0.5) [12] | 9.4 (0.7) [17] | 0.3026 | 1 |
| Similar | CHIT1 | Q13231 | 13.6 (1.2) [13] | 13.3 (0.8) [14] | 0.9271 | 1 |
| Unclear | A1BG | P04217 | 17.8 (0.4) [43] | 17.3 (0.3) [43] | <0.0001 | <0.0001 |
| Unclear | A1BG | P04217-2 | 17.2 (0.3) [43] | 16.8 (0.3) [43] | <0.0001 | <0.0001 |
| Unclear | A2M | P01023 | 16.5 (0.3) [43] | 16.7 (0.3) [43] | 0.0001 | 0.0926 |
| Unclear | ACSBG2 | Q5FVE4 | 20.9 (0.5) [43] | 21.8 (0.7) [43] | <0.0001 | <0.0001 |
| Unclear | AFM | P43652 | 15.4 (0.4) [43] | 15.1 (0.4) [43] | <0.0001 | 0.0001 |
| Unclear | AGRN | O00468-6 | 13.7 (0.9) [43] | 14.2 (0.6) [43] | 0.0018 | 1 |
| Unclear | AHSG | P02765 | 17.1 (0.6) [43] | 16.7 (0.5) [43] | <0.0001 | <0.0001 |
| Unclear | ALB | P02768 | 19.8 (0.3) [43] | 19.4 (0.3) [43] | <0.0001 | <0.0001 |
| Unclear | ALCAM | Q13740 | 14.9 (0.5) [43] | 15.4 (0.3) [43] | <0.0001 | 0.0001 |
| Unclear | ALDOA | P04075 | 14.2 (0.6) [43] | 14.9 (0.5) [43] | <0.0001 | 0.0005 |
| Unclear | ALDOC | P09972 | 14 (0.7) [43] | 14.9 (0.5) [43] | <0.0001 | <0.0001 |
| Unclear | AMBP | P02760 | 15.9 (0.5) [43] | 15.6 (0.4) [43] | <0.0001 | 0.0136 |
| Unclear | APCS | P02743 | 14.9 (0.7) [43] | 14.2 (0.6) [43] | <0.0001 | 0.0009 |
| Unclear | APLP1 | B7Z4G8 | 16.5 (0.7) [43] | 17.1 (0.3) [43] | <0.0001 | 0.0003 |
| Unclear | APOA1 | P02647 | 18.9 (0.4) [43] | 18.1 (0.4) [43] | <0.0001 | <0.0001 |
| Unclear | APOA2 | P02652 | 17.8 (0.5) [43] | 17.2 (0.6) [43] | <0.0001 | <0.0001 |
| Unclear | APOA4 | P06727 | 16.9 (0.6) [43] | 16.6 (0.5) [43] | <0.0001 | 0.003 |
| Unclear | APOC1 | K7ERI9 | 16 (0.8) [43] | 15.3 (0.6) [43] | <0.0001 | 0.0001 |
| Unclear | APOC3 | B0YIW2 | 17.2 (0.8) [43] | 16.2 (0.8) [43] | <0.0001 | <0.0001 |
| Unclear | APOD | C9JF17 | 17.7 (0.4) [43] | 17 (0.4) [43] | <0.0001 | <0.0001 |
| Unclear | APOE | P02649 | 17.5 (0.5) [43] | 17.7 (0.5) [43] | 0.0195 | 1 |
| Unclear | APOM | O95445 | 15.8 (0.6) [43] | 15.3 (0.6) [43] | 0.0001 | 0.0619 |
| Unclear | APP | P05067 | 14.7 (0.7) [43] | 15.2 (0.4) [43] | <0.0001 | 0.0004 |
| Unclear | ARPC4-TTLL3 | A0A0A6YYG9 | 16.7 (0.4) [43] | 16.5 (0.4) [43] | 0.0002 | 0.1058 |
| Unclear | ATP6AP1 | Q15904 | 14.7 (0.5) [43] | 15 (0.4) [43] | 0.0011 | 0.7316 |
| Unclear | AZGP1 | P25311 | 17.2 (0.3) [43] | 16.8 (0.3) [43] | <0.0001 | <0.0001 |
| Unclear | B2M | P61769 | 16.9 (0.3) [43] | 17.1 (0.4) [43] | 0.0484 | 1 |
| Unclear | B4GAT1 | O43505 | 16.5 (0.9) [43] | 17.1 (0.7) [43] | 0.0002 | 0.1314 |
| Unclear | BCAN | Q96GW7 | 15.5 (0.6) [43] | 16 (0.6) [43] | <0.0001 | 0.001 |
| Unclear | BTD | P43251 | 16.1 (0.4) [43] | 16.4 (0.3) [43] | <0.0001 | 0.0301 |
| Unclear | C1QA | P02745 | 14.6 (0.4) [43] | 14.4 (0.5) [43] | 0.0113 | 1 |
| Unclear | C1QB | D6R934 | 15.1 (0.5) [43] | 15.3 (0.4) [43] | 0.0004 | 0.2879 |
| Unclear | C2 | P06681 | 14.9 (0.4) [43] | 14.6 (0.3) [43] | <0.0001 | 0.0005 |
| Unclear | C3 | P01024 | 17.1 (0.2) [43] | 17 (0.2) [43] | <0.0001 | 0.0033 |
| Unclear | C5 | P01031 | 14.4 (0.5) [43] | 13.8 (0.4) [43] | <0.0001 | <0.0001 |
| Unclear | C6 | P13671 | 15.1 (0.5) [43] | 14.7 (0.3) [43] | <0.0001 | 0.0007 |
| Unclear | C7 | P10643 | 16.2 (0.4) [43] | 16 (0.4) [43] | 0.0001 | 0.0736 |
| Unclear | C8B | F5GY80 | 14.1 (0.3) [43] | 13.8 (0.3) [43] | <0.0001 | 0.0001 |
| Unclear | C9 | P02748 | 15.6 (0.5) [43] | 15.2 (0.4) [43] | <0.0001 | 0.0001 |
| Unclear | CADM4 | Q8NFZ8 | 15.9 (0.5) [43] | 16.4 (0.3) [43] | <0.0001 | <0.0001 |
| Unclear | CD44 | H0YD13 | 16.8 (0.6) [43] | 17.2 (0.4) [43] | 0.0003 | 0.2039 |
| Unclear | CDH2 | P19022 | 15.8 (0.4) [43] | 16.3 (0.3) [43] | <0.0001 | <0.0001 |
| Unclear | CFD | K7ERG9 | 16.6 (0.5) [43] | 16.4 (0.5) [43] | 0.0002 | 0.1006 |
| Unclear | CFH | P08603 | 15.7 (0.2) [43] | 15.4 (0.2) [43] | <0.0001 | <0.0001 |
| Unclear | CFI | E7ETH0 | 14.9 (0.3) [43] | 14.6 (0.3) [43] | <0.0001 | <0.0001 |
| Unclear | CHGA | P10645 | 16.1 (0.7) [43] | 16.7 (0.5) [43] | <0.0001 | 0.0011 |
| Unclear | CHGB | P05060 | 15.9 (0.7) [43] | 16.3 (0.5) [43] | 0.0001 | 0.0596 |
| Unclear | CHI3L1 | P36222 | 16 (0.5) [43] | 16.3 (0.5) [43] | <0.0001 | 0.0131 |
| Unclear | CHL1 | O00533 | 15.3 (0.7) [43] | 15.7 (0.5) [43] | <0.0001 | 0.0266 |
| Unclear | CLSTN1 | O94985 | 16.2 (0.7) [43] | 16.7 (0.4) [43] | <0.0001 | 0.0004 |
| Unclear | CNDP1 | Q96KN2 | 16.7 (0.6) [43] | 17.3 (0.5) [43] | <0.0001 | <0.0001 |
| Unclear | CNTN1 | Q12860 | 15.6 (0.7) [43] | 16.1 (0.4) [43] | 0.0001 | 0.053 |
| Unclear | COL1A1 | P02452 | 14.6 (0.6) [43] | 14.2 (0.5) [43] | 0.0001 | 0.0748 |
| Unclear | COL1A2 | A0A087WTA8 | 14.3 (0.4) [43] | 14.1 (0.3) [43] | 0.0002 | 0.1324 |
| Unclear | COL6A1 | A0A087X0S5 | 14.8 (0.5) [43] | 15 (0.4) [43] | 0.0047 | 1 |
| Unclear | CP | P00450 | 16.3 (0.3) [43] | 16.1 (0.3) [43] | <0.0001 | 0.0002 |
| Unclear | CPB2 | A0A087WSY5 | 15.2 (0.4) [43] | 15.1 (0.4) [43] | 0.0036 | 1 |
| Unclear | CPE | P16870 | 16.3 (0.8) [43] | 17 (0.5) [43] | <0.0001 | 0.0003 |
| Unclear | CPQ | Q9Y646 | 15.4 (0.8) [43] | 15.8 (0.5) [43] | 0.006 | 1 |
| Unclear | CSF1R | E9PEK4 | 15.2 (0.4) [43] | 15.5 (0.4) [43] | <0.0001 | 0.0006 |
| Unclear | CST3 | P01034 | 17.7 (0.4) [43] | 18.1 (0.3) [43] | <0.0001 | <0.0001 |
| Unclear | CTSD | A0A1B0GV23 | 16 (0.4) [43] | 16.9 (0.6) [43] | <0.0001 | <0.0001 |
| Unclear | CTSL | P07711 | 14.7 (0.4) [43] | 15.2 (0.5) [43] | <0.0001 | 0.0031 |
| Unclear | DAG1 | Q14118 | 15.6 (0.4) [43] | 15.8 (0.3) [43] | 0.0002 | 0.1477 |
| Unclear | DBI | A0A0A0MTI5 | 14.3 (0.4) [43] | 15.3 (0.6) [43] | <0.0001 | <0.0001 |
| Unclear | DKK3 | F6SYF8 | 16.1 (0.4) [43] | 16.5 (0.4) [43] | 0.0001 | 0.0617 |
| Unclear | EFEMP1 | A0A0U1RQV3 | 16.3 (0.4) [43] | 16.8 (0.4) [43] | <0.0001 | <0.0001 |
| Unclear | ENDOD1 | O94919 | 15.1 (0.4) [43] | 15.7 (0.3) [43] | <0.0001 | <0.0001 |
| Unclear | ENPP2 | E7EUF1 | 16.5 (0.4) [43] | 17.4 (0.6) [43] | <0.0001 | <0.0001 |
| Unclear | F12 | P00748 | 16.1 (0.6) [43] | 15.6 (0.5) [43] | <0.0001 | <0.0001 |
| Unclear | FAM3C | Q92520 | 15 (0.5) [43] | 15.6 (0.4) [43] | <0.0001 | <0.0001 |
| Unclear | FBLN1 | P23142 | 16.2 (0.4) [43] | 16.6 (0.4) [43] | <0.0001 | <0.0001 |
| Unclear | FBLN1 | B1AHL2 | 16.4 (0.4) [43] | 17 (0.4) [43] | <0.0001 | <0.0001 |
| Unclear | FCGR3A | A0A1W2PQB1 | 14.2 (1) [43] | 14.5 (0.7) [43] | 0.0357 | 1 |
| Unclear | FGA | P02671 | 14.9 (0.9) [43] | 14.1 (0.7) [43] | <0.0001 | 0.0004 |
| Unclear | GC | P02774 | 17 (0.3) [43] | 16.5 (0.3) [43] | <0.0001 | <0.0001 |
| Unclear | GM2A | P17900 | 15.9 (0.5) [43] | 16.4 (0.5) [43] | <0.0001 | 0.0185 |
| Unclear | GPR37L1 | O60883 | 14.2 (0.5) [43] | 14.5 (0.5) [43] | 0.0004 | 0.2598 |
| Unclear | GPX3 | A0A087X1J7 | 15.6 (0.4) [43] | 15.9 (0.5) [43] | 0.0004 | 0.2668 |
| Unclear | GSN | P06396 | 17 (0.3) [43] | 16.8 (0.2) [43] | 0.0076 | 1 |
| Unclear | HBA1 | P69905 | 16.3 (2.6) [43] | 17.9 (1.7) [43] | 0.002 | 1 |
| Unclear | HBB | P68871 | 16.1 (2.7) [43] | 17.7 (1.8) [43] | 0.0033 | 1 |
| Unclear | HEXB | P07686 | 14.2 (0.5) [43] | 14.8 (0.4) [43] | <0.0001 | <0.0001 |
| Unclear | HRG | P04196 | 16.2 (0.4) [43] | 15.7 (0.4) [43] | <0.0001 | <0.0001 |
| Unclear | HSPG2 | P98160 | 14.4 (0.3) [43] | 14.2 (0.4) [43] | 0.0187 | 1 |
| Unclear | HTRA1 | Q92743 | 14.3 (0.3) [43] | 14.6 (0.4) [43] | <0.0001 | 0.0036 |
| Unclear | ICOSLG | K4DIA0 | 15.6 (0.7) [43] | 16.1 (0.7) [43] | 0.0003 | 0.2155 |
| Unclear | IGFBP6 | P24592 | 18.4 (0.5) [43] | 17.8 (0.5) [43] | <0.0001 | 0.0001 |
| Unclear | IGHA1 | A0A286YEY1 | 17.7 (1) [43] | 17 (0.9) [43] | <0.0001 | <0.0001 |
| Unclear | IGHA2 | A0A286YEY5 | 17.6 (0.9) [43] | 17 (0.8) [43] | <0.0001 | 0.0001 |
| Unclear | IGHG1 | P01857 | 19.7 (0.5) [43] | 19.2 (0.4) [43] | <0.0001 | <0.0001 |
| Unclear | IGHG2 | P01859 | 19.8 (0.6) [43] | 19.3 (0.5) [43] | <0.0001 | <0.0001 |
| Unclear | IGHG3 | P01860 | 16.7 (1) [43] | 15.8 (0.8) [43] | <0.0001 | 0.0006 |
| Unclear | IGHV3-15 | A0A0B4J1V0 | 15.6 (0.6) [43] | 15.4 (0.6) [43] | 0.0039 | 1 |
| Unclear | IGHV3-30 | P01768 | 16.7 (0.5) [43] | 16.5 (0.4) [43] | 0.0007 | 0.4466 |
| Unclear | IGHV3-49 | A0A0A0MS15 | 16.5 (0.7) [43] | 16.3 (0.6) [43] | 0.0106 | 1 |
| Unclear | IGHV3-7 | P01780 | 17.1 (0.6) [43] | 16.8 (0.4) [43] | 0.001 | 0.6603 |
| Unclear | IGHV3-72 | A0A0B4J1Y9 | 16.5 (0.6) [43] | 16 (0.5) [43] | <0.0001 | 0.0084 |
| Unclear | IGHV3OR16-9 | A0A0B4J2B5 | 19.6 (0.9) [43] | 19.1 (0.7) [43] | <0.0001 | 0.0014 |
| Unclear | IGHV4-34 | P06331 | 14.9 (0.8) [43] | 14.5 (0.5) [43] | 0.0031 | 1 |
| Unclear | IGHV5-51 | A0A0C4DH38 | 15.8 (0.5) [43] | 15.5 (0.5) [43] | 0.0002 | 0.1079 |
| Unclear | IGKC | P01834 | 20.1 (0.5) [43] | 19.5 (0.5) [43] | <0.0001 | <0.0001 |
| Unclear | IGKV1-12 | A0A0C4DH73 | 16.9 (0.6) [43] | 16.4 (0.6) [43] | <0.0001 | <0.0001 |
| Unclear | IGKV1-5 | P01602 | 16.4 (0.6) [43] | 15.8 (0.7) [43] | <0.0001 | <0.0001 |
| Unclear | IGKV1-8 | A0A0C4DH67 | 16.3 (0.5) [43] | 15.8 (0.5) [43] | <0.0001 | <0.0001 |
| Unclear | IGKV1D-33 | P01593 | 17.3 (0.8) [43] | 16.8 (0.8) [43] | <0.0001 | <0.0001 |
| Unclear | IGKV2-29 | A2NJV5 | 15.9 (0.7) [43] | 15.5 (0.6) [43] | 0.0001 | 0.055 |
| Unclear | IGKV2D-24 | A0A075B6R9 | 16.4 (0.9) [43] | 16 (0.7) [43] | <0.0001 | 0.0289 |
| Unclear | IGKV3-20 | P01619 | 18.3 (0.7) [43] | 17.8 (0.5) [43] | <0.0001 | 0.0003 |
| Unclear | IGKV3D-11 | A0A0A0MRZ8 | 17.3 (0.5) [43] | 17.1 (0.5) [43] | 0.0018 | 1 |
| Unclear | IGKV3D-20 | A0A0C4DH25 | 17.9 (0.8) [43] | 17.5 (0.6) [43] | 0.0003 | 0.1936 |
| Unclear | IGKV4-1 | P06312 | 17.3 (0.6) [43] | 16.9 (0.5) [43] | 0.0002 | 0.1006 |
| Unclear | IGLC3 | P0DOY3 | 20.1 (0.7) [43] | 19.7 (0.6) [43] | 0.0001 | 0.0387 |
| Unclear | IGLL5 | A0A0B4J231 | 18 (0.7) [43] | 17.4 (0.5) [43] | <0.0001 | <0.0001 |
| Unclear | IGLV3-25 | P01717 | 15 (1.1) [43] | 14.3 (0.7) [43] | <0.0001 | 0.0044 |
| Unclear | IGLV3-9 | A0A075B6K5 | 15.2 (0.8) [43] | 14.4 (0.6) [43] | <0.0001 | <0.0001 |
| Unclear | IGSF8 | Q969P0 | 15.5 (0.4) [43] | 16.1 (0.3) [43] | <0.0001 | <0.0001 |
| Unclear | ITIH1 | P19827 | 15 (0.6) [43] | 14.7 (0.5) [43] | 0.0131 | 1 |
| Unclear | ITIH2 | P19823 | 15.1 (0.5) [43] | 14.9 (0.4) [43] | 0.0029 | 1 |
| Unclear | ITIH4 | Q14624 | 15.8 (0.5) [43] | 15.4 (0.4) [43] | <0.0001 | 0.0002 |
| Unclear | ITPR2 | Q14571 | 19 (0.8) [43] | 18.8 (0.8) [43] | 0.0336 | 1 |
| Unclear | KIAA1549L | H0YDE5 | 13.7 (0.8) [43] | 14.4 (0.6) [43] | <0.0001 | 0.0026 |
| Unclear | KLK6 | Q92876 | 16.8 (0.7) [43] | 17.6 (0.4) [43] | <0.0001 | <0.0001 |
| Unclear | KLKB1 | H0YAC1 | 13.9 (0.6) [43] | 13.4 (0.5) [43] | <0.0001 | 0.0001 |
| Unclear | KNG1 | P01042 | 17.2 (0.5) [43] | 16.6 (0.4) [43] | <0.0001 | <0.0001 |
| Unclear | LAMP2 | P13473 | 15.6 (0.7) [43] | 16.3 (0.5) [43] | <0.0001 | <0.0001 |
| Unclear | LDHA | P00338 | 13.1 (1.1) [43] | 13.6 (0.6) [43] | 0.0007 | 0.445 |
| Unclear | LDHB | P07195 | 14.2 (0.6) [43] | 14.9 (0.4) [43] | <0.0001 | 0.0007 |
| Unclear | LGALS3BP | Q08380 | 16.5 (0.4) [43] | 17 (0.3) [43] | <0.0001 | <0.0001 |
| Unclear | LRG1 | P02750 | 16.8 (0.4) [43] | 16.3 (0.4) [43] | <0.0001 | <0.0001 |
| Unclear | LSAMP | H3BLU2 | 14.9 (0.6) [43] | 15.4 (0.4) [43] | <0.0001 | <0.0001 |
| Unclear | LUM | P51884 | 15.9 (0.3) [43] | 15.5 (0.2) [43] | <0.0001 | <0.0001 |
| Unclear | MAN1C1 | Q9NR34 | 13.3 (0.6) [43] | 13.6 (0.5) [43] | 0.01 | 1 |
| Unclear | MMP2 | P08253 | 15.4 (0.2) [43] | 15.2 (0.3) [43] | 0.0011 | 0.7584 |
| Unclear | MOG | A0A0G2JHA9 | 13.7 (0.7) [43] | 14.4 (0.7) [43] | <0.0001 | 0.0037 |
| Unclear | NBL1 | A0A087WTY6 | 18.1 (0.7) [43] | 18.4 (0.6) [43] | 0.0029 | 1 |
| Unclear | NCAM1 | P13591 | 15.7 (0.5) [43] | 16.1 (0.4) [43] | <0.0001 | 0.0001 |
| Unclear | NPC2 | E7EMS2 | 16.4 (0.4) [43] | 16.9 (0.4) [43] | <0.0001 | <0.0001 |
| Unclear | NRCAM | C9JYY6 | 15.7 (0.7) [43] | 16.2 (0.4) [43] | <0.0001 | 0.0019 |
| Unclear | NUCB1 | Q02818 | 13.7 (0.4) [43] | 14 (0.4) [43] | <0.0001 | 0.024 |
| Unclear | OGN | P20774 | 16.7 (0.4) [43] | 16.1 (0.5) [43] | <0.0001 | <0.0001 |
| Unclear | ORM1 | P02763 | 19.5 (0.4) [43] | 19 (0.3) [43] | <0.0001 | <0.0001 |
| Unclear | ORM2 | P19652 | 18 (0.4) [43] | 17.6 (0.4) [43] | <0.0001 | <0.0001 |
| Unclear | PCOLCE | Q15113 | 16.4 (0.3) [43] | 16.2 (0.4) [43] | 0.0334 | 1 |
| Unclear | PCSK1N | Q9UHG2 | 15.1 (0.5) [43] | 15.6 (0.5) [43] | <0.0001 | <0.0001 |
| Unclear | PEBP1 | P30086 | 15.4 (0.4) [43] | 16.2 (0.4) [43] | <0.0001 | <0.0001 |
| Unclear | PEBP4 | Q96S96 | 15.7 (0.6) [43] | 16.3 (0.5) [43] | <0.0001 | <0.0001 |
| Unclear | PGLYRP2 | Q96PD5 | 14.8 (0.4) [43] | 14.5 (0.3) [43] | <0.0001 | 0.0021 |
| Unclear | PKM | P14618 | 13.9 (0.6) [43] | 14.8 (0.5) [43] | <0.0001 | <0.0001 |
| Unclear | PLG | P00747 | 16.3 (0.4) [43] | 15.9 (0.3) [43] | <0.0001 | <0.0001 |
| Unclear | PON1 | P27169 | 15.7 (0.6) [43] | 15.1 (0.6) [43] | <0.0001 | <0.0001 |
| Unclear | PPIB | P23284 | 15 (0.5) [43] | 15.2 (0.5) [43] | 0.049 | 1 |
| Unclear | PRDX2 | P32119 | 14.3 (1.7) [43] | 15.1 (0.9) [43] | 0.0059 | 1 |
| Unclear | PROS1 | P07225 | 14.9 (0.3) [43] | 15.2 (0.3) [43] | <0.0001 | 0.0054 |
| Unclear | PTGDS | P41222 | 20.8 (0.6) [43] | 20.9 (0.4) [43] | 0.03 | 1 |
| Unclear | PTPRZ1 | P23471 | 14.7 (0.6) [43] | 15.4 (0.4) [43] | <0.0001 | <0.0001 |
| Unclear | QSOX1 | O00391 | 14.1 (0.4) [43] | 14.3 (0.3) [43] | 0.0001 | 0.0877 |
| Unclear | RARRES2 | Q99969 | 15.9 (0.6) [43] | 16.1 (0.4) [43] | 0.0351 | 1 |
| Unclear | SAA4 | P35542 | 15.3 (0.7) [43] | 14.7 (0.7) [43] | <0.0001 | <0.0001 |
| Unclear | SCG2 | P13521 | 14.1 (0.7) [43] | 14.6 (0.5) [43] | <0.0001 | 0.0002 |
| Unclear | SCG3 | Q8WXD2 | 15.7 (0.6) [43] | 16.1 (0.5) [43] | <0.0001 | 0.004 |
| Unclear | SCG5 | P05408-2 | 15.6 (0.5) [43] | 16.2 (0.3) [43] | <0.0001 | <0.0001 |
| Unclear | SERPINA1 | P01009 | 18.5 (0.4) [43] | 18 (0.3) [43] | <0.0001 | <0.0001 |
| Unclear | SERPINA3 | P01011 | 17.2 (0.5) [43] | 17 (0.3) [43] | 0.0036 | 1 |
| Unclear | SERPINA4 | P29622 | 15.1 (0.4) [43] | 14.8 (0.3) [43] | <0.0001 | 0.0001 |
| Unclear | SERPINA6 | P08185 | 15.2 (0.4) [43] | 15 (0.3) [43] | 0.0025 | 1 |
| Unclear | SERPINA7 | P05543 | 14.9 (0.4) [43] | 14.8 (0.4) [43] | 0.0344 | 1 |
| Unclear | SERPINC1 | P01008 | 17.2 (0.3) [43] | 16.6 (0.3) [43] | <0.0001 | <0.0001 |
| Unclear | SERPIND1 | P05546 | 15.5 (0.3) [43] | 15.3 (0.3) [43] | 0.0001 | 0.0738 |
| Unclear | SERPINF1 | P36955 | 17.1 (0.3) [43] | 17.7 (0.5) [43] | <0.0001 | <0.0001 |
| Unclear | SERPINF2 | P08697 | 16.2 (0.4) [43] | 15.9 (0.3) [43] | <0.0001 | 0.0008 |
| Unclear | SERPING1 | P05155 | 16.5 (0.2) [43] | 16.3 (0.2) [43] | <0.0001 | 0.0001 |
| Unclear | SOD1 | P00441 | 15.7 (0.4) [43] | 16.2 (0.3) [43] | <0.0001 | <0.0001 |
| Unclear | SOD3 | P08294 | 15.4 (0.4) [43] | 16.1 (0.6) [43] | <0.0001 | <0.0001 |
| Unclear | SPARCL1 | Q14515 | 15 (0.5) [43] | 15.4 (0.3) [43] | <0.0001 | 0.0033 |
| Unclear | SPP1 | P10451 | 15.8 (0.3) [43] | 16.2 (0.3) [43] | <0.0001 | <0.0001 |
| Unclear | SULF2 | Q8IWU5 | 14.2 (0.8) [43] | 15 (0.4) [43] | <0.0001 | 0.0001 |
| Unclear | THY1 | E9PIM6 | 17 (0.8) [43] | 17.7 (0.6) [43] | <0.0001 | 0.0016 |
| Unclear | TPI1 | P60174 | 13.4 (0.7) [43] | 14.3 (0.6) [43] | <0.0001 | <0.0001 |
| Unclear | TPP2 | P29144 | 15.4 (0.7) [43] | 14.7 (0.9) [43] | <0.0001 | <0.0001 |
| Unclear | TTR | P02766 | 19.2 (0.6) [43] | 20 (0.8) [43] | <0.0001 | <0.0001 |
| Unclear | UBC | F5H265 | 15.7 (0.5) [43] | 16.3 (0.3) [43] | <0.0001 | <0.0001 |
| Unclear | VCAN | P13611 | 13.6 (0.5) [43] | 14.1 (0.3) [43] | 0.0001 | 0.0583 |
| Unclear | VGF | O15240 | 14.6 (0.8) [43] | 15.3 (0.5) [43] | <0.0001 | 0.0002 |
| Unclear | VIP | P01282 | 18.1 (0.9) [43] | 17.7 (0.5) [43] | 0.0057 | 1 |
| Unclear | VTN | P04004 | 17.2 (0.5) [43] | 16.8 (0.4) [43] | <0.0001 | 0.0001 |
| Unclear |  | B4E1Z4 | 15.2 (0.4) [43] | 14.7 (0.3) [43] | <0.0001 | <0.0001 |
| Unclear |  | A0A0J9YY99 | 15.5 (1.4) [43] | 15.2 (1.4) [43] | 0.0002 | 0.1351 |
| Unclear | APLP2 | Q06481 | 14.5 (0.8) [42] | 15 (0.5) [43] | 0.0005 | 0.3495 |
| Unclear | CALR | P27797 | 13.6 (0.4) [42] | 14 (0.4) [43] | <0.0001 | 0.0194 |
| Unclear | CD99L2 | Q8TCZ2 | 16.4 (0.7) [42] | 16.9 (0.5) [43] | <0.0001 | 0.0003 |
| Unclear | CDH13 | P55290 | 15.2 (0.5) [42] | 15.6 (0.4) [43] | <0.0001 | 0.0014 |
| Unclear | CLCNKB | A0A087X136 | 16.1 (0.6) [42] | 16.5 (0.7) [43] | 0.0002 | 0.1319 |
| Unclear | CPVL | Q9H3G5 | 14.2 (0.5) [42] | 14.8 (0.5) [43] | <0.0001 | <0.0001 |
| Unclear | CRTAC1 | A0A0C4DFP6 | 15.3 (0.5) [42] | 15.6 (0.4) [43] | <0.0001 | 0.0034 |
| Unclear | CTBS | Q01459 | 14.4 (0.4) [42] | 14.8 (0.5) [43] | <0.0001 | 0.0001 |
| Unclear | EPHA4 | E9PG71 | 15.2 (1.1) [42] | 15.5 (0.8) [43] | 0.0199 | 1 |
| Unclear | F9 | P00740 | 12.8 (0.6) [42] | 13.1 (0.6) [43] | 0.0248 | 1 |
| Unclear | FUCA1 | P04066 | 14.2 (0.6) [42] | 14.6 (0.6) [43] | <0.0001 | 0.003 |
| Unclear | GOT1 | P17174 | 14.4 (0.4) [42] | 15 (0.4) [43] | <0.0001 | <0.0001 |
| Unclear | GPR37 | O15354 | 15.3 (0.4) [42] | 15.9 (0.3) [43] | <0.0001 | <0.0001 |
| Unclear | IGFALS | P35858 | 14.5 (0.5) [42] | 14.1 (0.4) [43] | 0.0005 | 0.3649 |
| Unclear | IGHV2-26 | A0A0B4J1V2 | 13.6 (0.8) [43] | 13 (0.7) [42] | <0.0001 | <0.0001 |
| Unclear | IGKV2-28 | A0A075B6P5 | 16.3 (0.6) [42] | 15.7 (0.6) [43] | <0.0001 | <0.0001 |
| Unclear | ISLR | O14498 | 15.3 (0.2) [43] | 15 (0.3) [42] | <0.0001 | <0.0001 |
| Unclear | LMAN2 | D6RBV2 | 15.1 (0.5) [42] | 15.4 (0.4) [43] | 0.0021 | 1 |
| Unclear | NCAM2 | H9KV31 | 15 (0.4) [42] | 15.6 (0.4) [43] | <0.0001 | <0.0001 |
| Unclear | NEGR1 | Q7Z3B1 | 15.1 (1) [42] | 15.7 (0.6) [43] | <0.0001 | 0.0048 |
| Unclear | NELL2 | F8VVB6 | 15.1 (0.7) [42] | 15.7 (0.5) [43] | <0.0001 | 0.0001 |
| Unclear | NID1 | P14543 | 13.7 (0.5) [43] | 13.5 (0.4) [42] | 0.0133 | 1 |
| Unclear | NPTX1 | Q15818 | 15.5 (1) [42] | 16 (0.7) [43] | 0.0004 | 0.2581 |
| Unclear | NTM | Q9P121-4 | 14.9 (0.8) [42] | 15.3 (0.5) [43] | 0.0117 | 1 |
| Unclear | OAF | Q86UD1 | 15.2 (0.4) [42] | 14.8 (0.3) [43] | <0.0001 | 0.0011 |
| Unclear | PAM | P19021 | 15 (0.5) [42] | 15.4 (0.4) [43] | <0.0001 | 0.0021 |
| Unclear | PENK | P01210 | 14.9 (0.6) [42] | 15.5 (0.5) [43] | <0.0001 | 0.0013 |
| Unclear | PLXDC2 | Q6UX71 | 14.9 (0.4) [42] | 15.2 (0.4) [43] | <0.0001 | 0.0021 |
| Unclear | PRCP | P42785 | 14.9 (0.8) [42] | 15.2 (0.5) [43] | 0.0104 | 1 |
| Unclear | PRNP | A2A2V1 | 13.8 (0.7) [42] | 14.1 (0.5) [43] | 0.0018 | 1 |
| Unclear | PTPRD | P23468 | 14.1 (0.6) [42] | 14.7 (0.4) [43] | <0.0001 | <0.0001 |
| Unclear | PTPRG | P23470 | 14.9 (0.5) [42] | 15.2 (0.4) [43] | 0.0002 | 0.1009 |
| Unclear | SELENBP1 | Q13228 | 14.4 (0.8) [42] | 14.8 (0.4) [43] | 0.0214 | 1 |
| Unclear | WFIKKN2 | C9J6G4 | 15 (0.6) [42] | 15.4 (0.7) [43] | <0.0001 | 0.012 |
| Unclear | APLP1 | P51693-2 | 14.8 (0.8) [41] | 15.5 (0.7) [43] | <0.0001 | 0.0146 |
| Unclear | C1RL | Q9NZP8 | 14.1 (0.6) [43] | 13.6 (0.5) [41] | <0.0001 | 0.0004 |
| Unclear | CACNA2D1 | P54289 | 14.6 (0.5) [41] | 14.9 (0.4) [43] | <0.0001 | 0.012 |
| Unclear | CGREF1 | Q99674 | 13.5 (0.8) [41] | 13.9 (0.6) [43] | 0.0006 | 0.4134 |
| Unclear | CNTN2 | A0A1W2PQ11 | 15.7 (0.3) [41] | 16.2 (0.4) [43] | <0.0001 | <0.0001 |
| Unclear | CSF1 | P09603 | 14.2 (0.4) [41] | 14.6 (0.4) [43] | <0.0001 | 0.0163 |
| Unclear | CTSH | A0A087X0D5 | 14.8 (0.6) [41] | 15.4 (0.6) [43] | <0.0001 | <0.0001 |
| Unclear | HEXA | H3BP20 | 14.2 (0.7) [41] | 14.7 (0.5) [43] | 0.0001 | 0.0646 |
| Unclear | IGHG4 | A0A286YFJ8 | 16.1 (1.1) [42] | 15.8 (1) [42] | <0.0001 | 0.0072 |
| Unclear | MCAM | P43121 | 14.6 (0.8) [41] | 14.9 (0.6) [43] | 0.0088 | 1 |
| Unclear | MDH1 | P40925 | 14.9 (0.5) [41] | 15.6 (0.5) [43] | <0.0001 | <0.0001 |
| Unclear | NEO1 | Q92859 | 14.7 (0.5) [41] | 15.2 (0.4) [43] | <0.0001 | 0.0001 |
| Unclear | NFASC | O94856 | 14.4 (0.5) [41] | 15 (0.4) [43] | <0.0001 | <0.0001 |
| Unclear | NPDC1 | Q5SPY9 | 13.3 (0.5) [41] | 13.8 (0.5) [43] | <0.0001 | 0.0012 |
| Unclear | NPTXR | A0A1X7SBT7 | 15.4 (0.6) [41] | 15.7 (0.5) [43] | 0.0012 | 0.8239 |
| Unclear | OMG | P23515 | 15.5 (0.5) [41] | 16.2 (0.4) [43] | <0.0001 | <0.0001 |
| Unclear | PRELP | P51888 | 14.5 (0.4) [41] | 13.8 (0.5) [43] | <0.0001 | <0.0001 |
| Unclear | RNASET2 | A0A087WZM2 | 14.7 (0.4) [41] | 15 (0.4) [43] | <0.0001 | 0.0007 |
| Unclear | SEMA7A | O75326 | 14.2 (0.7) [41] | 14.8 (0.6) [43] | <0.0001 | 0.0001 |
| Unclear | SERPINI1 | Q99574 | 14.2 (0.5) [41] | 14.9 (0.5) [43] | <0.0001 | <0.0001 |
| Unclear | SEZ6L2 | A0A087WYL5 | 15.1 (0.7) [41] | 15.5 (0.5) [43] | 0.0004 | 0.2846 |
| Unclear | SIRPA | P78324 | 14.5 (0.6) [41] | 15 (0.6) [43] | <0.0001 | <0.0001 |
| Unclear | SOD2 | P04179 | 13.4 (0.4) [42] | 13.8 (0.3) [42] | <0.0001 | 0.0001 |
| Unclear | SPOCK1 | Q08629 | 13.7 (0.4) [41] | 14.3 (0.5) [43] | <0.0001 | <0.0001 |
| Unclear | TNXB | A0A140TA41 | 13.4 (0.5) [42] | 13.2 (0.5) [42] | 0.0033 | 1 |
| Unclear | TPP1 | O14773 | 14.3 (0.5) [41] | 14.9 (0.6) [43] | <0.0001 | <0.0001 |
| Unclear | VASN | Q6EMK4 | 14.5 (0.3) [41] | 14.7 (0.2) [43] | <0.0001 | 0.0148 |
| Unclear | ADGRB2 | A2A3C1 | 14.8 (0.7) [40] | 15.3 (0.6) [43] | <0.0001 | 0.0001 |
| Unclear | ART3 | E7ESB3 | 14.6 (0.4) [40] | 15 (0.4) [43] | <0.0001 | 0.0003 |
| Unclear | CUTA | O60888 | 14.7 (1.1) [40] | 15.2 (0.8) [43] | 0.0128 | 1 |
| Unclear | DSC2 | Q02487 | 14 (0.5) [41] | 14.2 (0.5) [42] | 0.0122 | 1 |
| Unclear | ERN1 | O75460 | 15.9 (1.2) [41] | 15.4 (1.3) [42] | 0.0084 | 1 |
| Unclear | EXTL2 | Q9UBQ6 | 14.6 (0.6) [40] | 15 (0.5) [43] | 0.0001 | 0.0355 |
| Unclear | FMOD | Q06828 | 14.5 (0.3) [41] | 13.8 (0.6) [42] | <0.0001 | <0.0001 |
| Unclear | FRZB | Q92765 | 14.8 (0.7) [41] | 14.4 (0.7) [42] | 0.0203 | 1 |
| Unclear | GSTP1 | P09211 | 14.2 (0.6) [40] | 15.2 (0.7) [43] | <0.0001 | <0.0001 |
| Unclear | LRRC4B | Q9NT99 | 14.4 (0.6) [40] | 15 (0.4) [43] | <0.0001 | <0.0001 |
| Unclear | MEGF8 | Q7Z7M0 | 14.2 (0.5) [40] | 14.5 (0.4) [43] | <0.0001 | 0.0112 |
| Unclear | NCAN | O14594 | 15 (0.5) [40] | 15.4 (0.4) [43] | <0.0001 | <0.0001 |
| Unclear | NRXN3 | A0A0U1RQC5 | 14.1 (0.6) [40] | 14.5 (0.5) [43] | <0.0001 | 0.0026 |
| Unclear | PIK3IP1 | Q96FE7-4 | 15 (0.5) [40] | 15.4 (0.4) [43] | <0.0001 | 0.0013 |
| Unclear | RNASE4 | P34096 | 13.9 (0.3) [40] | 13.6 (0.4) [43] | 0.0003 | 0.1699 |
| Unclear | SEZ6L | B0QYH4 | 14.2 (0.4) [40] | 14.4 (0.5) [43] | 0.0121 | 1 |
| Unclear | SPON1 | Q9HCB6 | 14 (0.7) [40] | 14.4 (0.6) [43] | 0.0002 | 0.1622 |
| Unclear | BASP1 | P80723 | 11.1 (0.9) [40] | 12.1 (0.8) [42] | <0.0001 | 0.0001 |
| Unclear | BPNT2 | Q9NX62 | 13.4 (0.6) [39] | 14 (0.6) [43] | <0.0001 | 0.0001 |
| Unclear | CADM1 | A0A087X0T8 | 15.5 (0.7) [39] | 16.1 (0.5) [43] | <0.0001 | 0.0014 |
| Unclear | CADM2 | Q8N3J6 | 14.6 (0.4) [39] | 14.9 (0.6) [43] | 0.0006 | 0.4248 |
| Unclear | CDH6 | D6RF86 | 12.9 (0.5) [40] | 13.2 (0.5) [42] | <0.0001 | 0.0073 |
| Unclear | DPP7 | Q9UHL4 | 13.8 (0.5) [39] | 14.1 (0.7) [43] | <0.0001 | 0.0102 |
| Unclear | GSTO1 | P78417 | 13.4 (0.8) [39] | 14 (0.6) [43] | 0.0007 | 0.4804 |
| Unclear | IL6ST | P40189 | 14.3 (0.4) [39] | 14.6 (0.4) [43] | <0.0001 | <0.0001 |
| Unclear | JCHAIN | D6RD17 | 15.1 (1.6) [42] | 14.4 (1.2) [40] | 0.0003 | 0.204 |
| Unclear | NRN1 | A0A087WWT2 | 15.2 (0.4) [39] | 15.5 (0.4) [43] | <0.0001 | 0.0197 |
| Unclear | OMD | Q99983 | 14.4 (0.5) [40] | 13.9 (0.7) [42] | <0.0001 | 0.0034 |
| Unclear | PEPD | P12955 | 12.3 (0.6) [42] | 12.6 (0.4) [40] | 0.0105 | 1 |
| Unclear | POMGNT1 | Q8WZA1 | 14.1 (0.4) [39] | 14.4 (0.4) [43] | 0.0012 | 0.8229 |
| Unclear | SHBG | I3L145 | 13.8 (0.5) [40] | 13.5 (0.3) [42] | 0.0001 | 0.0523 |
| Unclear | SORT1 | Q99523 | 13.8 (0.3) [39] | 14.2 (0.4) [43] | 0.0002 | 0.1623 |
| Unclear | SUSD5 | O60279 | 12.7 (0.5) [40] | 13 (0.4) [42] | 0.0006 | 0.4186 |
| Unclear | VCAM1 | P19320 | 13.8 (0.4) [40] | 13.7 (0.4) [42] | 0.0318 | 1 |
| Unclear | CPN2 | P22792 | 14.2 (0.7) [42] | 13.9 (0.6) [39] | 0.0107 | 1 |
| Unclear | CTSZ | Q9UBR2 | 14.9 (0.4) [39] | 15.5 (0.6) [42] | <0.0001 | 0.0001 |
| Unclear | IGF2 | P01344 | 14.8 (0.5) [41] | 14.4 (0.6) [40] | 0.0092 | 1 |
| Unclear | IGLV3-21 | P80748 | 15.4 (1.2) [41] | 14.7 (1) [40] | 0.0005 | 0.3661 |
| Unclear | MARCKS | P29966 | 11.3 (0.7) [39] | 11.8 (0.7) [42] | 0.0003 | 0.1964 |
| Unclear | NECTIN1 | Q15223 | 14.1 (0.5) [38] | 14.5 (0.3) [43] | <0.0001 | 0.0003 |
| Unclear | NPPC | P23582 | 13.9 (0.6) [38] | 14.1 (0.6) [43] | 0.0073 | 1 |
| Unclear | PLD3 | Q8IV08 | 14.5 (0.7) [38] | 14.9 (0.6) [43] | 0.0001 | 0.0498 |
| Unclear | PTPRS | Q13332 | 13.9 (0.7) [38] | 14.7 (0.3) [43] | <0.0001 | <0.0001 |
| Unclear | RGMB | J3KNF6 | 14.6 (0.7) [38] | 14.9 (0.6) [43] | 0.0003 | 0.1979 |
| Unclear | SPOCK3 | Q9BQ16 | 14.5 (0.4) [38] | 15.2 (0.6) [43] | <0.0001 | 0.0011 |
| Unclear | TMEM132A | Q24JP5 | 13.7 (0.5) [39] | 14.1 (0.5) [42] | 0.0016 | 1 |
| Unclear | VSTM2A | B5MCX6 | 14.5 (0.6) [38] | 14.8 (0.6) [43] | 0.0006 | 0.3958 |
| Unclear | CYCS | C9JFR7 | 12.8 (0.7) [38] | 13.3 (0.5) [42] | 0.0001 | 0.0788 |
| Unclear | FGFR2 | A0A0A0MR25 | 15.2 (0.8) [37] | 15.8 (0.6) [43] | 0.0001 | 0.0408 |
| Unclear | GDI2 | P50395 | 12.8 (1.1) [38] | 13.7 (0.7) [42] | 0.0001 | 0.0574 |
| Unclear | GGH | Q92820 | 14.4 (0.3) [38] | 14.6 (0.4) [42] | 0.0002 | 0.1146 |
| Unclear | H4C1 | P62805 | 12.8 (1.2) [38] | 13.5 (1.1) [42] | 0.0009 | 0.5841 |
| Unclear | IGKV3D-15 | A0A087WSY6 | 16.9 (0.8) [41] | 16.6 (0.6) [39] | 0.0019 | 1 |
| Unclear | SYT2 | Q8N9I0 | 18.1 (0.9) [41] | 17.5 (0.6) [39] | <0.0001 | 0.0327 |
| Unclear | TNR | Q92752 | 13.2 (0.4) [37] | 13.4 (0.5) [43] | 0.0011 | 0.7084 |
| Unclear | ACTA2 | P62736 | 14.7 (1) [36] | 15.5 (0.9) [43] | 0.0001 | 0.035 |
| Unclear | APOL1 | O14791 | 13.6 (0.7) [42] | 12.8 (0.8) [37] | <0.0001 | 0.0003 |
| Unclear | CACHD1 | A0A0A0MQY7 | 13.1 (0.5) [36] | 13.3 (0.4) [43] | 0.004 | 1 |
| Unclear | COLEC12 | Q5KU26 | 14.3 (0.6) [37] | 14.6 (0.4) [42] | 0.0102 | 1 |
| Unclear | ICAM5 | Q9UMF0 | 12.6 (0.8) [39] | 13.2 (0.6) [40] | <0.0001 | 0.0004 |
| Unclear | PLXNB1 | O43157 | 13 (0.5) [38] | 13.1 (0.5) [41] | 0.0307 | 1 |
| Unclear | PROC | E7END6 | 13 (0.5) [40] | 13.3 (0.5) [39] | 0.0004 | 0.2611 |
| Unclear | SEZ6 | Q53EL9 | 13.5 (0.7) [38] | 13.9 (0.6) [41] | 0.0009 | 0.5907 |
| Unclear | APOC2 | A0A024R0T9 | 14.9 (1.1) [42] | 14.4 (0.8) [36] | 0.0194 | 1 |
| Unclear | CTSS | P25774 | 13.8 (0.5) [38] | 14.1 (0.4) [40] | 0.0045 | 1 |
| Unclear | GNPTG | Q9UJJ9 | 15.3 (0.4) [35] | 15.4 (0.4) [43] | 0.0134 | 1 |
| Unclear | IGHV3-38 | A0A0C4DH36 | 15.2 (0.6) [38] | 15 (0.5) [40] | 0.0053 | 1 |
| Unclear | IL31RA | Q8NI17 | 23.6 (0.4) [41] | 24.4 (0.6) [37] | <0.0001 | <0.0001 |
| Unclear | MAG | P20916 | 12.9 (0.4) [37] | 13.5 (0.5) [41] | <0.0001 | <0.0001 |
| Unclear | MAN2A2 | P49641 | 13.8 (0.6) [35] | 14.1 (0.5) [43] | 0.0054 | 1 |
| Unclear | TRBV7-2 | A0A1B0GXF2 | 15.6 (0.5) [39] | 15.1 (0.5) [39] | <0.0001 | <0.0001 |
| Unclear | ENPP4 | Q9Y6X5 | 12.6 (0.4) [36] | 13 (0.5) [41] | <0.0001 | 0.0005 |
| Unclear | EPDR1 | Q9UM22 | 13 (0.8) [34] | 13.7 (0.6) [43] | <0.0001 | 0.0137 |
| Unclear | FAM3C | C9JP35 | 14.5 (0.5) [35] | 14.9 (0.5) [42] | 0.0001 | 0.0335 |
| Unclear | FUCA2 | Q9BTY2 | 14.2 (1) [39] | 14.6 (0.9) [38] | 0.0012 | 0.8204 |
| Unclear | GALNT2 | Q10471 | 13.7 (0.4) [36] | 13.9 (0.3) [41] | 0.0048 | 1 |
| Unclear | SORCS3 | Q9UPU3 | 12.9 (0.8) [36] | 13.5 (0.8) [41] | 0.0007 | 0.4719 |
| Unclear | CNDP1 | J3KRP0 | 17 (0.8) [33] | 17.5 (0.7) [43] | 0.0003 | 0.1729 |
| Unclear | CTSF | Q9UBX1 | 14.7 (0.3) [33] | 14.8 (0.3) [43] | 0.0046 | 1 |
| Unclear | KRT1 | P04264 | 14.8 (1.5) [34] | 15.3 (1.7) [42] | 0.0311 | 1 |
| Unclear | LRP1 | Q07954 | 13.5 (0.6) [33] | 13.7 (0.4) [43] | 0.014 | 1 |
| Unclear | ADAM29 | Q9UKF5 | 15.6 (0.8) [34] | 15.1 (0.7) [41] | 0.0147 | 1 |
| Unclear | CACNA2D1 | P54289-2 | 14.6 (0.5) [34] | 15.1 (0.5) [41] | <0.0001 | 0.0001 |
| Unclear | FSTL4 | Q6MZW2 | 12.8 (0.8) [35] | 13.2 (0.7) [40] | 0.0005 | 0.3025 |
| Unclear | FXYD6 | Q9H0Q3 | 14.3 (0.7) [32] | 15.2 (0.6) [43] | <0.0001 | 0.013 |
| Unclear | GRIA4 | G3V164 | 12.7 (0.6) [36] | 12.9 (0.5) [39] | 0.0177 | 1 |
| Unclear | ITM2B | Q9Y287 | 12.7 (1.2) [32] | 13.9 (1.5) [43] | 0.0024 | 1 |
| Unclear | SKP1 | E5RJR5 | 13.4 (0.6) [33] | 14.2 (0.6) [42] | <0.0001 | 0.0158 |
| Unclear | ATRN | O75882 | 13.9 (0.4) [33] | 14 (0.3) [41] | 0.0202 | 1 |
| Unclear | NAGLU | P54802 | 13 (0.8) [33] | 13.4 (0.8) [41] | 0.0087 | 1 |
| Unclear | ADGRB3 | O60242 | 11.1 (0.6) [35] | 11.4 (0.6) [38] | 0.0001 | 0.0407 |
| Unclear | GOLM1 | Q8NBJ4 | 13.2 (0.5) [30] | 13.5 (0.5) [43] | <0.0001 | 0.0104 |
| Unclear | SYNE3 | G3V533 | 14.6 (0.9) [40] | 14.5 (0.8) [33] | 0.011 | 1 |
| Unclear | CLN5 | A0A024R644 | 13.3 (1) [35] | 13.9 (0.6) [37] | 0.0147 | 1 |
| Unclear | HGFAC | D6RAR4 | 14.3 (0.5) [40] | 13.9 (0.5) [32] | 0.0013 | 0.8939 |
| Unclear | PLXNB2 | O15031 | 13.6 (0.6) [30] | 13.9 (0.6) [42] | 0.0093 | 1 |
| Unclear | TGFBR3 | Q03167 | 12.5 (0.6) [32] | 12.9 (0.7) [40] | 0.0271 | 1 |
| Unclear | B4GALT1 | P15291 | 14.1 (0.5) [36] | 13.9 (0.5) [35] | 0.0259 | 1 |
| Unclear | CARTPT | Q16568 | 13.1 (0.8) [29] | 13.7 (0.6) [42] | 0.0001 | 0.0865 |
| Unclear | CNTFR | P26992 | 12.8 (1.1) [30] | 13.1 (0.8) [41] | 0.0479 | 1 |
| Unclear | IGLV8-61 | A0A075B6I0 | 15 (1.1) [38] | 14.8 (0.8) [33] | 0.0127 | 1 |
| Unclear | ADIPOQ | Q15848 | 12.2 (0.6) [36] | 11.8 (0.5) [34] | <0.0001 | 0.0152 |
| Unclear | ASAH1 | A0A1B0GTM3 | 14.5 (0.4) [29] | 14.8 (0.5) [41] | <0.0001 | <0.0001 |
| Unclear | PCDH1 | Q08174 | 13 (0.6) [29] | 13.5 (0.5) [41] | <0.0001 | 0.0004 |
| Unclear | SHISA6 | Q6ZSJ9 | 13.8 (0.4) [31] | 14.1 (0.5) [39] | 0.0001 | 0.0776 |
| Unclear | HYOU1 | A0A087X054 | 13.9 (0.4) [28] | 14.3 (0.3) [41] | <0.0001 | 0.0037 |
| Unclear | PGAM1 | P18669 | 13.9 (1) [26] | 14.7 (0.8) [43] | 0.0003 | 0.1685 |
| Unclear | PGK1 | P00558 | 13.4 (1.1) [29] | 14 (0.8) [40] | 0.008 | 1 |
| Unclear | PODXL2 | Q9NZ53 | 11.8 (1.1) [34] | 12.7 (0.8) [35] | 0.0002 | 0.1199 |
| Unclear | SLITRK3 | O94933 | 13.4 (0.5) [36] | 13.6 (0.5) [33] | 0.0002 | 0.1119 |
| Unclear | TXN | P10599 | 16 (0.8) [26] | 16.3 (0.4) [43] | 0.0333 | 1 |
| Unclear | FAT2 | Q9NYQ8 | 12.8 (0.6) [32] | 12.9 (0.7) [36] | 0.0286 | 1 |
| Unclear | IGKV2D-29 | A0A075B6S2 | 14.5 (0.6) [34] | 14 (0.6) [33] | 0.0003 | 0.2185 |
| Unclear | IGLV3-10 | A0A075B6K4 | 15.2 (1.3) [33] | 14.7 (0.8) [34] | 0.0022 | 1 |
| Unclear | ADGRL3 | E7EN28 | 13 (1.2) [27] | 13.7 (0.9) [39] | 0.001 | 0.6416 |
| Unclear | IGFBP5 | P24593 | 12 (0.6) [26] | 12.5 (0.7) [40] | 0.0053 | 1 |
| Unclear | PRDX1 | Q06830 | 14.6 (1.8) [25] | 15.7 (1.1) [41] | 0.0083 | 1 |
| Unclear | TNFRSF21 | O75509 | 14.4 (0.4) [27] | 14.8 (0.4) [39] | 0.0003 | 0.182 |
| Unclear | IGLV6-57 | P01721 | 14.5 (0.8) [33] | 13.6 (0.8) [32] | <0.0001 | 0.0049 |
| Unclear | MST1 | G3XAK1 | 13 (0.4) [31] | 12.6 (0.6) [34] | 0.0001 | 0.0433 |
| Unclear | PSAP | C9JIZ6 | 13.6 (0.9) [23] | 14.3 (0.9) [42] | 0.0041 | 1 |
| Unclear | ZNF511-PRAP1 | H7BY64 | 13.8 (0.3) [23] | 14.1 (0.6) [42] | 0.0494 | 1 |
| Unclear | CCDC93 | F8W9X7 | 14.2 (0.4) [28] | 14.5 (0.5) [36] | 0.0047 | 1 |
| Unclear | CTSA | P10619 | 12.8 (0.4) [26] | 13.4 (0.6) [38] | <0.0001 | 0.0308 |
| Unclear | RTN4RL2 | Q86UN3 | 14.1 (0.6) [25] | 14.6 (0.5) [39] | 0.0001 | 0.0435 |
| Unclear | MANBA | O00462 | 13.1 (0.5) [26] | 13.3 (0.4) [37] | 0.021 | 1 |
| Unclear | PRKCSH | K7ELL7 | 14.6 (0.8) [25] | 15.1 (0.5) [38] | 0.0012 | 0.7774 |
| Unclear | MIF | P14174 | 14.9 (0.8) [24] | 15.5 (0.6) [38] | 0.006 | 1 |
| Unclear | CBLN1 | P23435 | 12.3 (0.9) [27] | 12.6 (0.8) [34] | 0.003 | 1 |
| Unclear | GLDN | Q6ZMI3 | 13.5 (0.6) [20] | 14.1 (0.7) [41] | 0.0001 | 0.0539 |
| Unclear | IGLV7-46 | A0A075B6I9 | 15.4 (1.1) [36] | 15.1 (0.9) [25] | 0.0002 | 0.113 |
| Unclear | GPLD1 | P80108 | 14 (0.6) [34] | 13.5 (0.5) [26] | 0.0003 | 0.2331 |
| Unclear | MIA | Q16674 | 16 (0.5) [35] | 15.1 (0.5) [25] | <0.0001 | <0.0001 |
| Unclear | PRDX6 | P30041 | 14 (1.4) [20] | 14.7 (1.2) [40] | 0.0299 | 1 |
| Unclear | SPINT2 | K7EM91 | 12.6 (0.6) [27] | 12.9 (0.5) [33] | 0.0016 | 1 |
| Unclear | ACYP2 | P14621 | 11.5 (0.5) [20] | 12.1 (0.6) [39] | 0.0002 | 0.1641 |
| Unclear | CCN3 | P48745 | 14.2 (0.5) [31] | 13.5 (0.4) [28] | <0.0001 | <0.0001 |
| Unclear | IGSF21 | Q96ID5 | 14.9 (0.6) [21] | 14 (1.7) [38] | 0.0256 | 1 |
| Unclear | NTNG1 | Q9Y2I2 | 11.7 (0.8) [25] | 12.2 (0.8) [34] | 0.0034 | 1 |
| Unclear | HSPA8 | P11142 | 13 (1.8) [15] | 14.3 (1.1) [42] | 0.0043 | 1 |
| Unclear | IGHV1-2 | P23083 | 15.4 (0.8) [29] | 15.1 (0.5) [28] | 0.0486 | 1 |
| Unclear | IGKV1-27 | A0A075B6S5 | 15.8 (0.8) [27] | 15.3 (0.7) [30] | 0.019 | 1 |
| Unclear | PTPRK | E9PGC5 | 12 (0.4) [24] | 12.4 (0.5) [33] | 0.0021 | 1 |
| Unclear | CAMK2A | Q9UQM7 | 13.6 (0.6) [24] | 14.3 (0.7) [32] | 0.0004 | 0.2625 |
| Unclear | CRP | P02741 | 14.4 (0.8) [31] | 14.1 (1) [25] | 0.04 | 1 |
| Unclear | CSPG5 | A0A087WUT8 | 14.3 (0.4) [19] | 14.7 (0.4) [36] | <0.0001 | 0.0274 |
| Unclear | SEC23IP | Q9Y6Y8 | 13.5 (0.5) [19] | 14.1 (0.7) [36] | 0.0008 | 0.5193 |
| Unclear | TUBA1B | P68363 | 12.2 (1.8) [13] | 16.6 (1.6) [42] | 0.0001 | 0.0778 |
| Unclear | SERPINA1 | A0A0G2JRN3 | 18 (2.9) [30] | 18 (2.3) [24] | 0.028 | 1 |
| Unclear | CETP | P11597 | 14.9 (0.5) [20] | 15.2 (0.4) [32] | 0.0065 | 1 |
| Unclear | PCSK1 | P29120 | 11.7 (2.7) [19] | 13.1 (2.2) [33] | 0.1195 | 1 |
| Unclear | IGKV2-40 | A0A087WW87 | 13.8 (0.8) [26] | 13.5 (0.6) [25] | 0.0002 | 0.1131 |
| Unclear | MMRN2 | Q9H8L6 | 14.2 (0.4) [17] | 14.4 (0.3) [34] | 0.0465 | 1 |
| Unclear | C1QTNF3-AMACR | E9PGA6 | 13.7 (0.8) [20] | 12.2 (2.3) [30] | 0.0212 | 1 |
| Unclear | CAT | P04040 | 15.3 (1.4) [27] | 14.4 (1.5) [23] | 0.0425 | 1 |
| Unclear | CCHCR1 | A0A0G2JHN4 | 14.4 (1.6) [20] | 13.6 (2.1) [30] | 0.0144 | 1 |
| Unclear | PTPRF | P10586 | 12 (1.4) [17] | 13.4 (1.3) [32] | 0.0061 | 1 |
| Unclear | ABHD14B | Q96IU4 | 12.2 (1) [22] | 12.5 (0.7) [26] | 0.0104 | 1 |
| Unclear | ATP1B1 | P05026 | 12 (0.9) [10] | 13.3 (1.2) [38] | 0.0001 | 0.0659 |
| Unclear | PROZ | P22891 | 13.6 (0.9) [26] | 13.1 (0.5) [22] | 0.0135 | 1 |
| Unclear | CHST15 | Q7LFX5 | 13.5 (0.5) [20] | 13 (0.5) [25] | 0.0126 | 1 |
| Unclear | RGMA | A0A0A0MTQ4 | 14.6 (0.4) [17] | 14.8 (0.6) [28] | 0.0252 | 1 |
| Unclear | CNTNAP2 | Q9UHC6 | 12.8 (0.5) [18] | 13.5 (0.7) [26] | 0.0312 | 1 |
| Unclear | ISLR2 | Q6UXK2 | 16.9 (1.8) [19] | 14.5 (2.4) [25] | 0.0012 | 0.8324 |
| Unclear | CANT1 | Q8WVQ1 | 12.9 (0.5) [11] | 13.4 (0.3) [32] | 0.0019 | 1 |
| Unclear | HBG2 | P69892 | 14 (2.4) [18] | 14.5 (2.3) [25] | 0.1886 | 1 |
| Unclear | TAC1 | P20366 | 14.1 (0.8) [16] | 14.7 (0.6) [27] | 0.015 | 1 |
| Unclear | DPP6 | E9PF59 | 12.4 (0.7) [15] | 13 (0.7) [27] | 0.0045 | 1 |
| Unclear | KRT14 | P02533 | 11 (2.4) [17] | 11.8 (2.9) [23] | 0.1487 | 1 |
| Unclear | IGKV1D-13 | A0A0B4J2D9 | 16.1 (1.1) [18] | 15.4 (0.7) [21] | 0.0067 | 1 |
| Unclear | H2BC12 | O60814 | 13.9 (0.5) [12] | 14.4 (1) [26] | 0.003 | 1 |
| Unclear | HYAL1 | Q12794 | 16.2 (1.1) [18] | 15.8 (0.7) [19] | 0.0006 | 0.4311 |
| Unclear | COMP | G3XAP6 | 12 (1.1) [14] | 11.3 (0.9) [18] | 0.033 | 1 |
| Unclear | KNG1 | P01042-2 | 8 (0.7) [15] | 7.6 (0.7) [15] | 0.034 | 1 |
| Unclear | KHSRP | A0A087WTP3 | 13.7 (0.4) [17] | 13.5 (0.3) [12] | 0.0089 | 1 |
| Unclear | GC | D6RF35 | 17.6 (0.8) [15] | 17.6 (0.5) [13] | 0.0096 | 1 |
